# Self-propelled gas nanomotor-integrated microneedles for melanoma therapy: Dual-action in situ eradication and metastatic suppression

**DOI:** 10.1016/j.mtbio.2025.102122

**Published:** 2025-07-21

**Authors:** Chungchi Lee, Shanghui Huang, Huiling Liu, Xinyue He, Yifan Hao, Lizhi Zeng, Yuhan Li, Zijin Lv, Yiyang Xu, Rui Guo

**Affiliations:** Key Laboratory of Biomaterials of Guangdong Higher Education Institutes, Key Laboratory of Regenerative Medicine of Ministry of Education, Guangdong Provincial Engineering and Technological Research Centre for Drug Carrier Development, Department of Biomedical Engineering, Jinan University, Guangzhou, 510632, China

**Keywords:** Photothermal therapy, Immunotherapy, Microneedle patch, Melanoma

## Abstract

Skin cancer, particularly malignant melanoma, is one of the most prevalent cancers globally, affecting millions annually. Traditional treatments like excision, chemotherapy, and immunotherapy often fail due to their invasiveness, toxicity, and poor drug delivery efficiency caused by skin barriers. Microneedles (MNs) offer a minimally invasive alternative, directly penetrating the skin to deliver drugs to tumor sites while minimizing side effects. However, their penetration depth is limited, and they do not address metastatic lesions. This study developed a gas motor-driven multi-responsive microneedle patch that significantly enhances subcutaneous drug penetration through a gas propulsion mechanism, greatly expanding the drug's diffusion range. Combined with photothermal therapy (PTT) and chemotherapy, this approach rapidly kills melanoma cells locally, induces immunogenic cell death (ICD), and uses anti-PD-1 antibodies (aPD-1) targeting immune checkpoints to enhance efficacy, overcome tumor immune escape, and activate systemic immune responses, achieving remote tumor suppression through local treatment only.

## Graphical abstract

**Figure:**
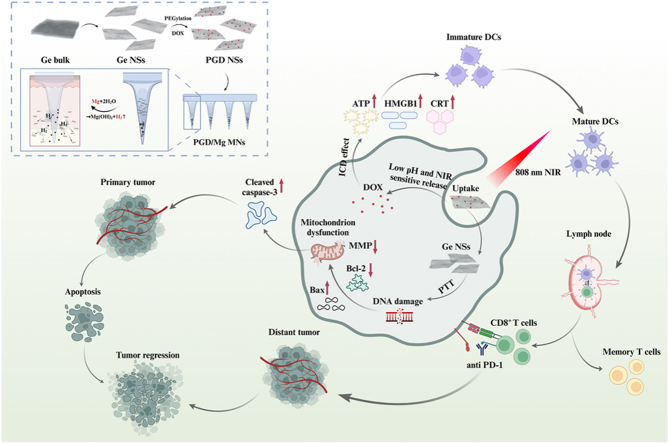

## 1 Introduction

Skin cancer is one of the most common cancer types, with continuously rising incidence rates. It ranks among the top five cancers globally, affecting millions of patients each year [[1], [2], [3]]. Malignant melanoma, one of the most aggressive and lethal types of skin cancer, has a notably low five-year survival rate for advanced-stage patients [4,5]. Conventional treatments for melanoma mainly include surgical resection, chemotherapy, immunotherapy, and topical administration [6]. However, surgical resection can lead to significant skin defects, incomplete tumor removal, and a high risk of local recurrence [7,8]. Chemotherapy has limited efficacy and systemic administration often causes multi-organ toxicity [9,10]. Traditional topical formulations such as ointments, gels, and patches [11] exhibit poor delivery efficiency and limited penetration into skin tumor tissues because of the stratum corneum barrier, resulting in inadequate therapeutic effects and elevated recurrence risk [12]. Microneedles (MNs), as a novel minimally invasive transdermal delivery approach, can penetrate the skin barrier to create microchannels, enabling direct drug entry into tumor tissues [[13], [14], [15]]. Moreover, MNs can avoid stimulation of deeper neural tissues, significantly reducing discomfort and skin damage [16,17]. This minimally invasive property makes MNs a promising delivery modality, especially for localized cancer treatment. Currently, dissolvable microneedle patches with rapid drug release capability have been extensively utilized in skin cancer therapies [18]. Within these dissolvable MNs, drugs diffuse freely through the skin upon dissolution of the matrix materials. Although drug release kinetics can be controlled by adjusting matrix composition, once the microneedles fully dissolve, the drugs rely solely on passive diffusion, making it challenging to regulate penetration depth and distribution range [19,20]. Additionally, simply increasing MN length to achieve deeper drug penetration is impractical due to unnecessary skin irritation and potential damage. These limitations significantly constrain the effectiveness of traditional dissolvable MNs, particularly when deeper tissue penetration or delivery into thickened skin is required [21].

Photothermal therapy (PTT) is considered an effective modality for cancer treatment [22,23]. However, due to the heterogeneous distribution of heat within tumor tissues, PTT alone often fails to completely eradicate cancer cells, and residual cells may subsequently cause recurrence and metastasis [24,25]. Specific chemotherapeutics, such as doxorubicin (DOX) and oxaliplatin, have been demonstrated to induce immunogenic cell death (ICD) effectively [26]. ICD promotes dendritic cell (DC) recruitment and activation by exposing calreticulin (CRT) on the tumor cell surface and releasing intracellular damage-associated molecular patterns (DAMPs), thereby enhancing tumor antigen uptake by DCs [27,28]. This process further stimulates the host immune system for sustained antitumor effects [29]. However, tumor cells frequently evade immune surveillance by upregulating PD-L1 expression, thereby promoting PD-1 expression on CD8^+^ T cells and facilitating immune escape [30]. Consequently, ICD-induced antitumor immune responses are compromised, limiting systemic antitumor immunity and failing to effectively suppress distant tumor lesions [31]. Immune checkpoint blockade (ICB), by disrupting interactions between immune checkpoints and their receptors, has emerged as a promising approach for cancer therapy with durable efficacy [[32], [33], [34], [35], [36], [37]]. Recently, antibodies targeting PD-1 [38,39] and PD-L1 [40,41] have been approved for lung cancer, melanoma, and liver cancer treatment. Nevertheless, the response rates for immunotherapy remain limited to approximately 20 %–30 %, significantly constraining its broader application and further development [[42], [43], [44], [45]]. Previous studies have indicated that induction of ICD could substantially enhance the efficacy of ICB therapies [46], as ICD closely associates with activation of tumor-specific T cells, thus synergizing effectively with immune checkpoint antibodies [47].

**Scheme 1:**
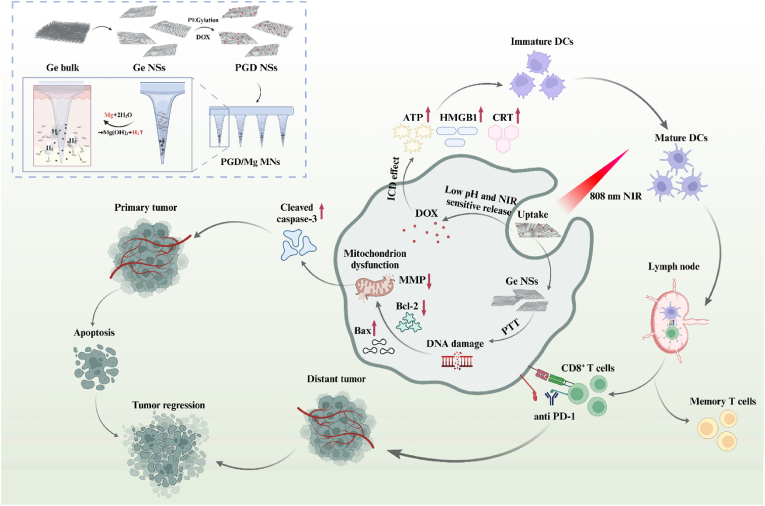
The preparation of PGD/Mg MNs, the gas-driven microneedle-mediated drug release mechanism, and the therapeutic strategy combining PGD/Mg MNs (+) with aPD-1/Mg MNs for effective antitumor treatment and systemic immune activation, enabling distant tumor suppression solely through localized in situ therapy. Created in BioRender. https://BioRender.com/undefined.

In summary, current microneedle technology exhibits limited drug delivery depth and lacks therapeutic efficacy against distant tumor lesions, representing significant drawbacks in melanoma treatment. To address this, we designed a multi-responsive microneedle patch, magnesium (Mg) particles are ingeniously utilized as gas-driven motors, which generate hydrogen gas (H_2_) through rapid reaction with interstitial fluid. This gas propulsion mechanism provides an active driving force within the microneedles, significantly enhancing drug diffusion and penetration into subcutaneous and tumor tissues. Modified Ge nanosheets loaded at microneedle tips possess excellent drug-loading capacity and exhibit multi-stimulus responsiveness (pH and NIR-triggered drug release). Under near-infrared (NIR) irradiation, their exceptional photothermal effect rapidly damages mitochondria, kills melanoma cells, and releases chemotherapeutics, thus inducing ICD, promoting dendritic cell maturation, and facilitating immune cell accumulation in tumor tissues to continuously eradicate residual melanoma cells. Moreover, combining this approach with PD-1/PD-L1 blockade effectively promotes CD8^+^ T cell infiltration into the tumor microenvironment, thereby triggering a robust systemic immune response capable of suppressing the progression of distant tumors. By overcoming the inherent limitations of passive diffusion in conventional dissolvable microneedles, this self-propelling system facilitates deep, precise, and efficient in situ delivery, thereby offering a promising strategy for effective local therapy and robust suppression of distant tumor lesions (Scheme 1).

## 2 Results and discussion

### 2.1 Synthesis and characterization of PGD NSs

**Fig. 1:**
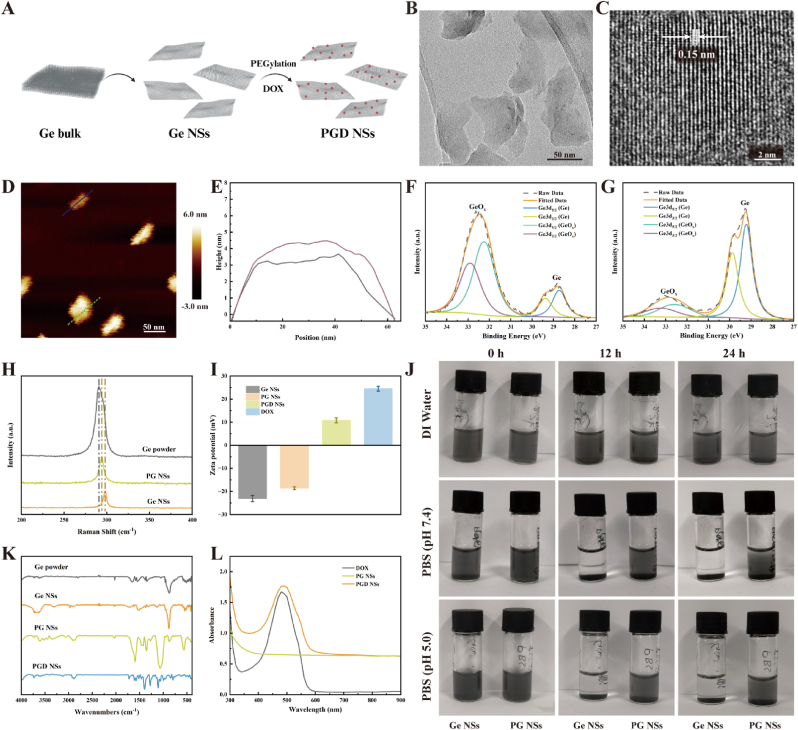
Synthesis and Characterization of PGD NSs. Created in BioRender. https://BioRender.com/undefined. (A) Schematic of PGD NSs synthesis. (B) HRTEM image and (C) lattice arrangement of Ge NSs. (D) AFM image of Ge NSs. (E) Ge NS thickness measured from the AFM image. (F) XPS spectra of Ge NSs and (G) PG NSs. (H) Raman spectra of Ge powder, Ge NSs, and PG NSs. (I) Zeta potential analysis of Ge NSs, PG NSs, PGD NSs, and DOX. (J) Dispersion behavior of Ge NSs and PG NSs in DI water, PBS at pH 7.4, and PBS at pH 5.0 after 0, 12, and 24 h. (K) FT-IR spectra of Ge powder, Ge NSs, PG NSs, and PGD NSs. (L) UV–vis spectra of PG NSs, PGD NSs, and DOX.

Ge nanosheets (Ge NSs) were prepared by exfoliating bulk Ge powder through a liquid-phase top-down approach (Fig. 1A). The morphological features of the prepared Ge NSs were comprehensively investigated by employing atomic force microscopy (AFM) and high-resolution transmission electron microscopy (HRTEM). HRTEM images revealed that the average lateral size of the Ge NSs was approximately 50 nm (Fig. 1B), with a lattice spacing of 0.15 nm (Fig. 1C). The hydrodynamic diameter, measured by dynamic light scattering (DLS), was approximately 68.6 nm (Fig. S1A), confirming the two-dimensional sheet-like structure and atomic-level ordered arrangement of Ge NSs. AFM analysis showed that the average thickness of the Ge NSs was about 3.5 nm (Fig. 1D and E), indicating their ultrathin nature and uniform distribution, reflecting good electronic transparency and distinct 2D properties [48].

he chemical composition of Ge NSs was further elucidated by X-ray photoelectron spectroscopy (XPS) (Fig. 1F and S2A). The Ge3d peaks observed in the XPS spectra of Ge NSs at binding energies of 29.9 eV and 29.3 eV are indicative of the crystalline germanium phase [49]. Additionally, the characteristic peak of germanium oxide (GeOx) could be attributed to surface oxidation caused by exposure to air during the synthesis process. Owing to electronic shielding, Ge NSs frequently undergo aggregation and precipitation in solutions with high ionic strength. To address this limitation and significantly enhance their stability and dispersibility in physiological media, we functionalized the Ge NSs surface with DSPE-PEG (5k). This modification effectively minimized particle aggregation by introducing steric hindrance and hydrophilic interactions, markedly improving their compatibility and maintaining colloidal stability under biologically relevant conditions. As shown in Fig. 1G and S2B, the XPS spectra of PEG-modified germanium nanosheets (PG NSs) demonstrate a significant reduction in the characteristic peaks of GeOx, indicating that the DSPE-PEG modification effectively reduces the likelihood of surface oxidation. Moreover, after 24 h of incubation in PBS at pH 5.0, PG NSs maintained good dispersion and high stability (Fig. 1J), demonstrating their ability to remain stable in the acidic tumor microenvironment.

The chemical composition of Ge NSs, both prior to and following DSPE-PEG modification, was analyzed by Fourier-transform infrared spectroscopy (FTIR) to confirm successful surface functionalization. As shown in Fig. 1K and S3, the characteristic absorption peaks observed at 2916 cm^−1^ and 2875 cm^−1^ correspond to symmetric stretching vibrations of C–H bonds. Additionally, the distinct absorption peak appearing between 1598 and 1596 cm^−1^ can be assigned to the stretching vibration of the C=O functional group. Furthermore, the crystalline structures of both pristine Ge NSs and PG NSs were thoroughly characterized using Raman spectroscopy and X-ray diffraction (XRD) analyses, confirming their structural integrity and phase composition. The Raman spectrum of Ge NSs exhibited a slight redshift compared to bulk Ge powder, indicating a transition from bulk to nanosheet form [50,51] (Fig. 1G). The Raman peaks of PG NSs showed a slight blueshift compared to Ge NSs, indicating minimal change in thickness after PEG grafting [[52], [53], [54]]. XRD analysis of Ge NSs before and after PEG grafting showed consistent diffraction peaks with the standard Ge card (JCPDS 04–0545) (Fig. S4). Furthermore, no significant change in the hydration or particle size of Ge NSs after PEG grafting was observed (Fig. S1B). These results confirm the successful preparation of both Ge NSs and PG NSs.

As shown in Fig. 1I, after PEG grafting, the surface negative charge of Ge NSs was significantly reduced, indicating that PEG modification effectively shields the surface charge and enhances the stability of the material. PG NSs show strong positive charge after loading of the chemotherapeutic drug DOX (PGD NSs), suggesting their ability to effectively adsorb negatively charged DOX molecules through electrostatic interactions [55]. Centrifugation experiments revealed that the DOX-loaded PGD NSs formed a characteristic red precipitate, while no noticeable color change was observed in the supernatant (Fig. S5). The average hydrodynamic diameter of PGD NSs was approximately 105 nm with a uniform size distribution (Fig. S1C). Furthermore, serum stability assays demonstrated that PGD NSs maintained stable particle size and good dispersion after incubation in 10 % fetal bovine serum (FBS) for 24 h, confirming their excellent *in vitro* stability (Fig. S1D). FTIR spectra showed characteristic peaks of DOX in the 1700-1100 cm^−1^ region for PGD NSs (Fig. 1K and S4), and UV–vis spectra exhibited a prominent DOX peak at 480 nm (Fig. 1L), collectively confirming the successful loading of DOX.

### 2.2 Drug release and photothermal properties of PGD NSs

**Fig. 2:**
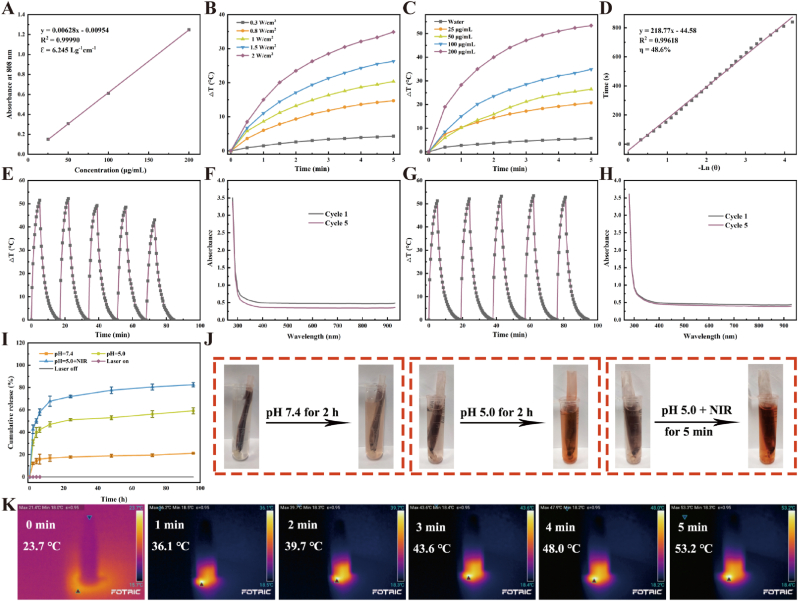
Drug release and photothermal properties of PGD NSs. (A) Extinction coefficient of PG NSs at 808 nm. (B) Temperature increase curves of PG NSs solutions at different concentrations (0, 25, 50, 100, 200 μg/mL) under NIR irradiation (808 nm, 2 W/cm^2^). (C) Temperature increase curves of PGD NSs solution (200 μg/mL) under 808 nm NIR irradiation at different powers (0.3, 0.8, 1, 1.5, 2 W/cm^2^). (D) Photothermal stability of PG NSs solution under NIR laser irradiation (808 nm). (E) Ge NSs and (G) PG NSs photostability. UV–vis NIR absorbance of (F) Ge NSs solution and (H) PG NSs at the 1st and 5th cycles. (I, J) DOX release behavior in PBS solutions at different pH values with and without NIR irradiation (808 nm, 1 W/cm^2^) (n = 3). (K) Temperature increase image of PGD NSs under NIR irradiation (808 nm, 1W/cm^2^) for 5 min.

The concentration of Ge NSs was precisely quantified using inductively coupled plasma mass spectrometry (ICP-MS). Notably, the absorbance at 808 nm (A808) demonstrated a strong linear relationship with the concentration of Ge NSs, underscoring the reliable optical responsiveness of the nanosheets (Fig. 2A). A greater extinction coefficient signifies enhanced light absorption capability of the material [56]. In accordance with the Beer-Lambert law, the extinction coefficient of PG NSs at 808 nm was determined to be 6.245 L g^−1^ cm^−1^, markedly surpassing those of several representative photothermal agents (PTAs), including nano Fe_3_O_4_ (5.13 L g^−1^ cm^−1^) [57] and reduced graphene oxide (rGO) (3.6 L g^−1^ cm^−1^) [58], thereby demonstrating its superior light absorption capability. To comprehensively assess the photothermal performance of PG NSs, solutions at different concentrations were subjected to irradiation with an 808 nm NIR laser. The results demonstrated a clear dependency of photothermal performance on both nanosheet concentration and laser power intensity (Fig. 2B and C). Remarkably, a maximum temperature elevation (ΔT) of 53.4 °C was recorded at a concentration of 200 μg/mL under irradiation of 2 W/cm^2^ (Fig. S6), highlighting the outstanding photothermal capabilities of the PG NSs. The photothermal conversion efficiency (η) of PG NSs was determined using previously established methodologies [53], resulting in an η value of 48.6 % (Fig. 2D). This value is markedly higher than that of numerous conventional PTAs. For instance, the η value of the heterostructure bismuth selenide and tungsten selenide nanosheets (BW) is 40.75 % [59], the η value of Pd SAzyme is 33.98 % [60], and the η value of Fe-rGO micromotor is 27 % [61]. This relatively high photothermal conversion efficiency makes PG NSs a highly promising photothermal agent.

Additionally, photothermal stability remains a critical factor influencing the practical application of PTAs. To systematically investigate this, we subjected both PG NSs and pristine Ge NSs solutions to repeated cycles of heating under 808 nm NIR irradiation for 5 min, followed by natural cooling to ambient temperature, while continuously monitoring their temperature variations. After five cycles, PG NSs still displayed a significant photothermal effect during the temperature increase (Fig. 2E and F), while the photothermal effect of Ge NSs significantly decreased under the same conditions (Fig. 2G and H), indicating that PEGylation improves photothermal stability. We further studied the controlled drug release behavior of PGD NSs under different conditions. In an environment with pH 5.0, 56.3 % of DOX was released over 72 h, while only 19.5 % was released at pH 7.4. The observed phenomenon is attributable to the increased hydrophilicity of DOX under acidic conditions due to protonation of its amino groups, demonstrating that PGD NSs exhibit effective pH responsiveness within the acidic tumor microenvironment. Additionally, PGD NSs exhibited significant photothermal responsiveness. After NIR laser irradiation, the cumulative release of DOX at pH 5.0 significantly increased to 80.5 % over 72 h (Fig. 2I and J). After drug loading, the photothermal capability of PGD NSs was re-evaluated, and the results showed that under 808 nm NIR irradiation, PGD NSs still heated up to 53.2 °C within 5 min, indicating that drug loading had minimal impact on the photothermal capability of PG NSs (Fig. 2K).

Based on the aforementioned experimental results, the PGD NSs drug delivery system is capable of responding to both low pH and NIR laser stimuli *in vitro*. The synergistic effect of these two stimuli enables precise control of drug release. This dual-responsive mechanism provides strong support for the controllability and therapeutic efficacy of drug delivery.

### 2.3 Performance characterization of PGD MNs

**Fig. 3:**
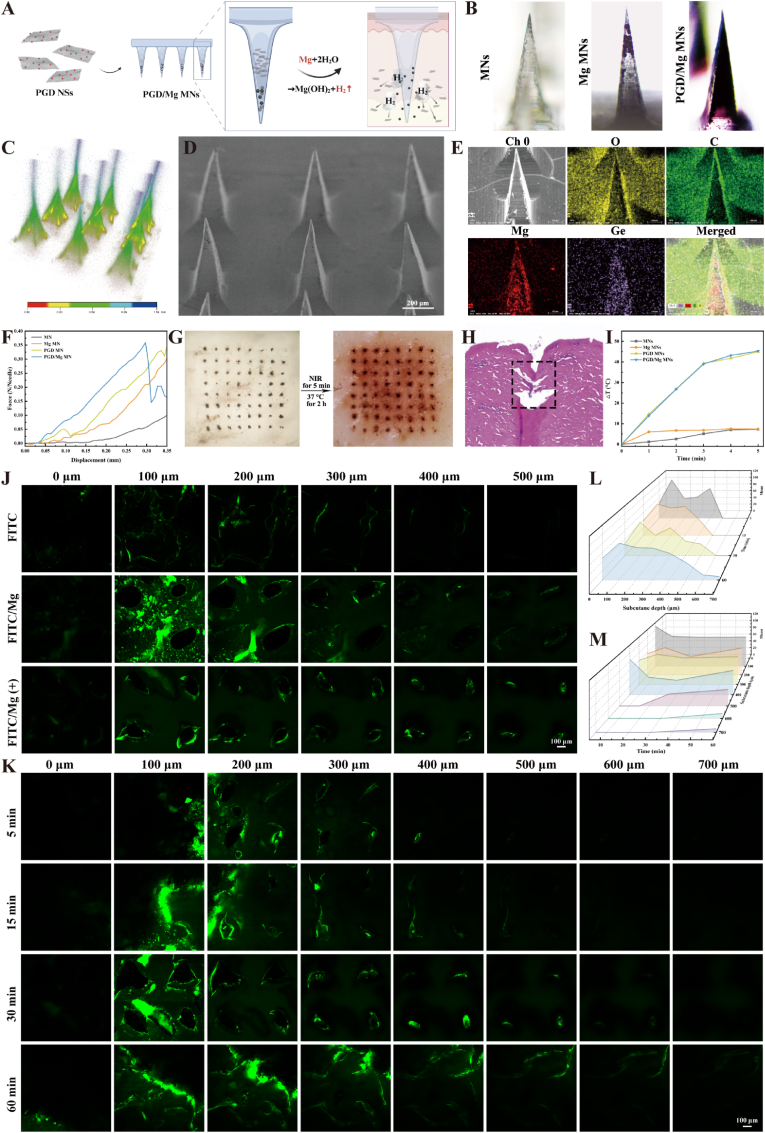
Performance characterization of PGD MNs. (A) Schematic of the microneedle interior and gas motor. Created in BioRender. https://BioRender.com/undefined. (B) Microscopic images of microneedle tips loaded with different drugs. (C) 3D CLSM image of aPD-1 MNs. (D) SEM images of PGD/Mg MNs. (E) EDX elemental analysis of PGD/Mg MNs. (F) Mechanical performance testing of microneedles loaded with different drugs. (G) Images of PGD/Mg MNs penetrating ex vivo skin followed by 808 nm NIR irradiation for 5 min and incubation at 37 °C for 2 h. (H) H&E images of PGD/Mg MNs penetrating ex vivo skin. (I) Temperature increases curves of microneedles loaded with different drugs under NIR irradiation (808 nm, 2 W/cm^2^). (J) Depth of *in vitro* drug release from microneedles loaded with different drugs with or without NIR irradiation (808 nm, 1.5 W/cm^2^, 5 min). (K) *In vitro* drug release depth of PGD/Mg MNs after NIR irradiation (808 nm, 1.5 W/cm^2^, 5 min) at 5, 15, 30, and 60 min. (L, M) *In vitro* drug release time and depth analysis based on (K).

We first loaded magnesium (Mg) metal particles at the tips of the microneedles and then loaded PGD NSs, followed by filling DEX as the microneedle base material to prepare PGD/Mg MNs (Fig. 3A). Fig. 3C and S7 show the 8 × 8 microneedle array, clearly demonstrating that multiple microneedles with a height of 800 μm can efficiently load drugs and active protein components while maintaining a robust hard structure and sharp tips. The digital microscope image of a single microneedle tip, magnified in Fig. 3B, further illustrates the ability of a single microneedle to carry both active particles and therapeutic payloads.

The scanning electron microscopy (SEM) analysis of PGD/Mg MNs revealed minimal alteration in surface morphology following the incorporation of nanomaterials (Fig. 3D). Energy-dispersive X-ray spectroscopy (EDX) analysis validated the uniform encapsulation of Mg and Ge microparticles within the microneedle matrix (Fig. 3E). Mechanical puncture tests performed with a universal testing machine demonstrated that the integration of nanomaterials significantly improved the mechanical strength and penetration capability of the microneedle tips (Fig. 3F). While the toughness of the material decreased upon loading with Mg, resulting in tip fracture, the puncture force reached approximately 0.36 N per needle, which is much higher than the 0.098 N per needle required to penetrate human skin [62]. This suggests that the microneedles can withstand the high compressive forces exerted by the skin during penetration without undergoing deformation [63].

The puncture ability of the microneedles after drug encapsulation was further validated through porcine skin puncture simulation (Fig. 3G and H). The results showed that PGD MNs were able to successfully penetrate the porcine skin, and the skin surface after puncture showed distinct micro-needle matrix holes and residual metal particles. Additionally, significant DOX release was observed 2 h after puncture. The experiment also verified the photothermal performance of the drug-loaded microneedles. The results indicated that the encapsulation of the microneedles and the loading of Mg particles did not significantly affect the photothermal capability of PGD NSs when inserted into porcine skin (Fig. 3I–S8).

To explore whether the gas generated by the interaction of Mg particles with bodily fluids could drive the drug deeper into the skin, and whether photothermal effects could enhance this driving effect, FITC was used as a marker for investigation. Confocal laser scanning microscopy (CLSM) revealed that the inclusion of Mg particles substantially enhanced the tissue penetration of the dye (Fig. 3J), indicating a significant increase in the dye's penetration range. This expansion is attributed to the dissolution of the microneedle tips exposing the Mg particles, which react instantaneously with the interstitial fluid within the skin, leading to the rapid formation of hydrogen bubbles that exert a localized force, thereby enhancing the subcutaneous penetration of the dye [64]. Furthermore, after NIR irradiation, the penetration depth of the dye was further increased, likely due to the enhanced dissolution of the microneedle tips under photothermal conditions, which accelerated the release of FITC [65]. To explore the drug release behavior of the FITC/Mg (+) group more thoroughly, different time points were set for observation, and fluorescence intensity was quantitatively analyzed (Fig. 3K–M). The confocal images showed that the drug remained primarily in the superficial epidermis (0–400 μm) within the first 15 min, and after 30 min, the drug reached deeper skin tissues (500–600 μm). The integration of gas propulsion with PTT markedly augments the depth of drug penetration, significantly enhancing its subcutaneous delivery efficacy.

### 2.4 *In vitro* antitumor capacity of PGD MNs

**Fig. 4:**
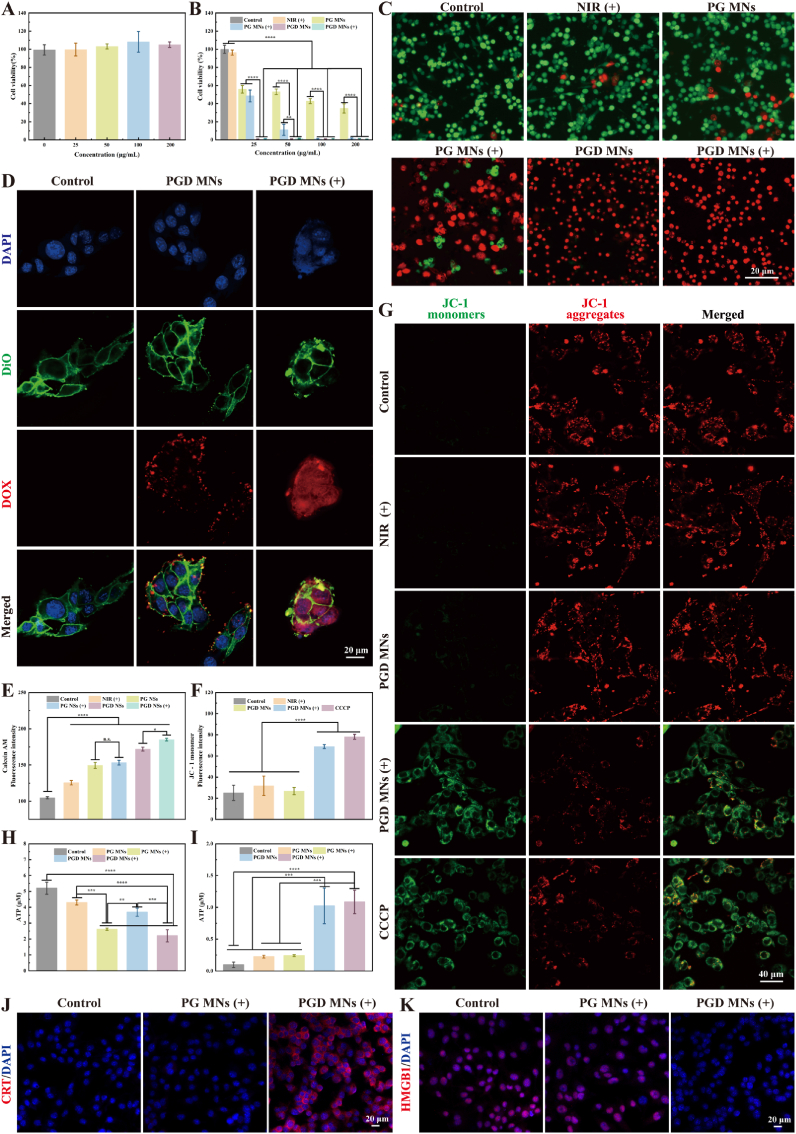
*In vitro* antitumor capacity of PGD MNs. (A) Cytotoxicity of microneedles loaded with different concentrations of PG NSs after co-culturing with L929 cells for 24 h. (B) Viability of B16 cells following treatment with microneedles loaded with various drugs, with or without NIR irradiation. (808 nm, 1 W/cm^2^, 5 min). (C) Fluorescent images of live (calcein-AM, green) and dead (PI, red) B16 cells after treatment with microneedles loaded with different drugs and NIR exposure. (D) Phagocytosis of DOX by B16 cells after various treatments. (DAPI nuclear stain, blue; DiO cell membrane, green; DOX, red) (E) Quantification of live/dead cell staining based on (C). (F) Quantification of membrane potential staining based on (G). (G) Alterations in the mitochondrial membrane potential of B16 cells following treatment with microneedles, with or without PGD NSs, in the presence or absence of NIR irradiation. (JC-1 monomers, green; JC-1 aggregates, red) (H) Intracellular ATP levels in B16 cells. (I) Extracellular ATP release levels in B16 cells. (J) Representative immunofluorescence images illustrating CRT exposure on the surface of B16 cells following different treatment conditions. (DAPI nuclear stain, blue; CRT, red) (K) Representative immunofluorescence images depicting HMGB1 release from B16 cells following different treatment conditions. (DAPI nuclear stain, blue; HMGB1, red).

We first evaluated the biocompatibility of PG NSs *in vitro* using L929 cells, which showed excellent cytocompatibility (Fig. 4A). Subsequently, we assessed the photothermal cytotoxicity of PG MNs toward B16 cells. Upon co-incubation with PG MNs followed by exposure to an 808 nm NIR laser for 5 min, cell viability markedly decreased in a concentration-dependent manner. Notably, when PGD NSs loaded with DOX were used at a concentration as low as 25 mg/mL, the viability of B16 cells dramatically reduced to below 1 % (Fig. 4B).

To further confirm the outstanding photothermal effect of PGD MNs on B16 cancer cells, a live/dead cell staining experiment was conducted. The results indicated that under 808 nm NIR irradiation, the majority of B16 cells lost their viability. In contrast, cells treated with NIR alone or PG MNs alone showed negligible cell death (Fig. 4C–E). Combining the drug release behavior, the experimental results revealed that when PGD NSs reached the tumor site, the acidic microenvironment of the tumor and NIR irradiation together triggered the release of DOX, allowing small molecule drugs to penetrate deep into the tumor, thereby enhancing tumor penetration through NIR stimulation. Fig. 4D illustrates that, in the absence of NIR irradiation, DOX fluorescence remained localized to the cell membrane. However, after NIR induction, stronger DOX fluorescence signals were observed inside the tumor spheres, including deeper core regions, indicating that NIR stimulation significantly promoted the phagocytosis and absorption of the drug by B16 cells, verifying the excellent photothermal-induced penetration of PGD NSs.

Mitochondrial dysfunction is considered one of the major mechanisms of cell death [66], and DNA damage and reduced mitochondrial membrane potential are both markers of apoptosis [67]. We examined the anticancer mechanism of PGD MNs activated by NIR irradiation through JC-1 staining to monitor mitochondrial membrane potential (MMP) alterations. JC-1 dye forms red fluorescent aggregates in healthy mitochondria with high MMP, whereas mitochondrial damage reduces MMP, resulting in green fluorescent monomers of JC-1. The experimental results showed that cells treated with NIR laser alone or PGD MNs alone exhibited fluorescence intensities almost identical to the control group. Following combined treatment with PGD MNs and 808 nm NIR irradiation, a marked increase in green fluorescence was observed, signifying severe mitochondrial damage. This shift reflects a pronounced decline in MMP and a substantial decrease in intracellular adenosine triphosphate (ATP) levels, highlighting the profound impact of NIR-activated PGD MNs on cellular energy dynamics (Fig. 4F–H). Cellular TEM images further revealed evident mitochondrial swelling in the PGD MNs group (Fig. S9, indicated by red arrows), accompanied by features such as cristae reduction and disruption of the double membrane. Overall analysis suggests that during PTT, PGD MNs induce mitochondrial dysfunction through localized high temperatures, accompanied by changes in MMP, leading to cell death.

It has been reported that the chemotherapeutic drug DOX can induce ICD, which promotes dying tumor cells to release DAMPs and activate the immune system. Therefore, we analyzed three DAMPs, including CRT, ATP, and high mobility group box 1 (HMGB1), to investigate ICD induced by PGD MNs. Confocal fluorescence images showed that the CRT exposure level on the plasma membrane of B16 cells treated with PGD MNs (+) was the highest (Fig. 4J), while the intracellular level of HMGB1 decreased, indicating that after drug induction, HMGB1 was stimulated to be secreted extracellularly (Fig. 4K). Additionally, the PGD MNs (+) group showed the highest ATP release levels, suggesting a stronger ICD-inducing effect. This mechanism strongly supports the antitumor potential of PGD MNs in photothermal combinatorial drug therapy.

### 2.5 *In vivo* antitumor capacity of PGD/mg MNs and aPD-1 MNs/Mg in combination

**Fig. 5:**
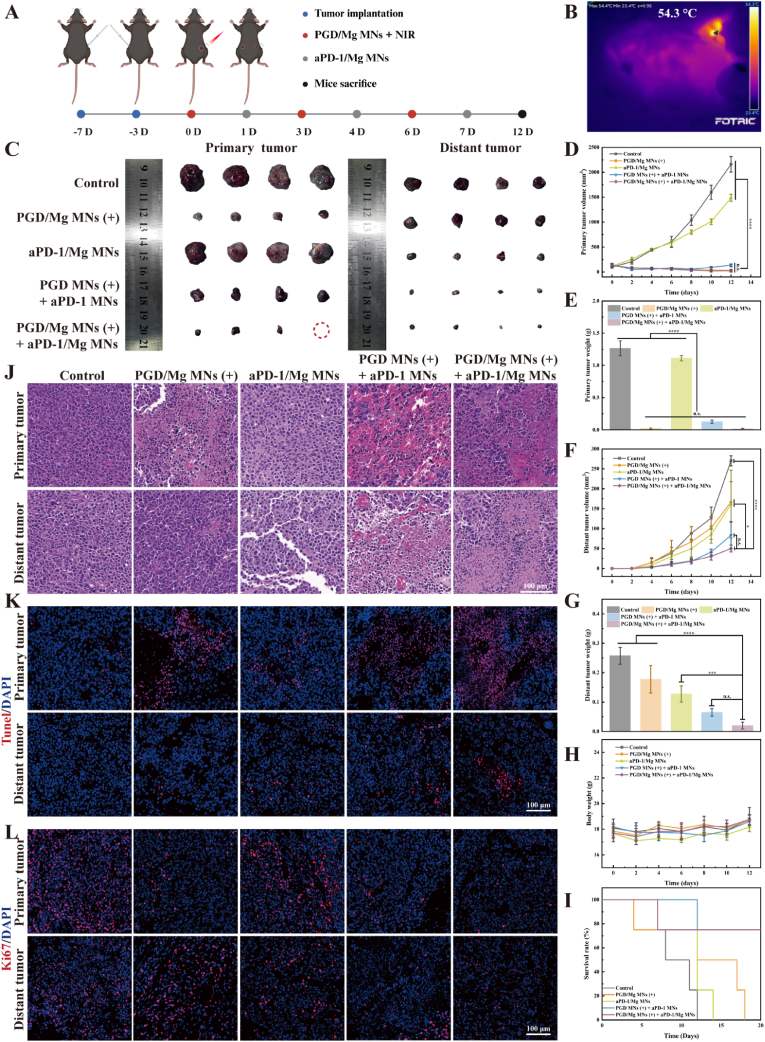
*In vivo* antitumor capacity of PGD/Mg MNs and aPD-1 MNs/Mg in combination. **(**A) Experimental design of animal treatments. Created in BioRender. https://BioRender.com/undefined. (B) *In vivo* photothermal images of PGD/Mg MNs under NIR irradiation (808 nm, 1.5 W/cm^2^, 5 min). (C) Images of primary and distal tumors under different treatments. (n = 4). Tumor volume curves of (D) primary tumors and (F) distal tumors under different treatments. (n = 4). Tumor weight of (E) primary tumors and (G) distal tumors under different treatments. (n = 4). (H) Changes in mouse body weight during treatment. (n = 4). (I) Survival statistics of mice within 20 days post-treatment. (n = 5). (J) H&E, (K) Tunel (DAPI nuclear stain, blue; Tunel, red), and (L) Ki67 (DAPI nuclear stain, blue; Ki67, red) staining of tumor tissues extracted from mice on day 12.

Next, we investigated the *in vivo* antitumor effects of PGD/Mg MNs combined with anti-PD-1 microneedles (aPD-1/Mg MNs) in C57BL/6 mice. In the experiment, B16 melanoma cells were subcutaneously implanted into both flanks of mice. The treatment protocol involved administering PGD/Mg MNs on days 0, 3, and 6, and aPD-1/Mg MNs on days 1, 4, and 7 (Fig. 5A). NIR irradiation at 808 nm and an intensity of 1.5 W/cm^2^ was applied for 5 min. During this procedure with PGD/Mg MNs (+), the temperature at the tumor site was maintained at approximately 53 °C throughout the NIR exposure (Fig. 5B and S10).

Experimental findings revealed that the PGD/Mg MNs (+) treatment markedly suppressed the growth of the primary tumor on the right side compared to both the untreated control and the group treated only with PGD MNs (+) (Fig. 5C–E). Similarly, the growth of the left-side tumor (distal tumor) was also significantly suppressed in the PGD/Mg MNs (+) combined with aPD-1/Mg MNs treatment group (Fig. 5C–F). Subsequent assessments showed that both primary and distal tumors were significantly lighter in the group receiving combined treatment compared to those in the untreated group (Fig. 5E–G), Additionally, this treatment regimen considerably prolonged survival, with the mice achieving a median survival time of 20 days (Fig. 5I).

Histological evaluations, including hematoxylin and eosin (H&E) staining and immunofluorescence, demonstrated that the treatment with PGD/Mg MNs (+) effectively induced tumor cell death (Fig. 5J–L). This result indicates that PGD/Mg MNs promoted the transport of PGD NSs to the tumor site and effectively killed tumor cells under NIR irradiation. Notably, the significant suppression of distal tumors in the PGD/Mg MNs (+) combined with aPD-1/Mg MNs treatment group suggests that the treatment activated a systemic antitumor immune response.

Furthermore, none of the treatment groups exhibited significant weight loss, suggesting that the soluble microneedle-based PGD NSs and aPD-1 microneedles did not induce apparent systemic toxicity (Fig. 5H). H&E staining of major organ tissues revealed no significant inflammatory infiltration or other pathological abnormalities (Fig. S11). The red blood cell, white blood cell, and platelet counts, along with other routine hematological parameters, remained within the normal range across all treatment groups (Fig. S12). Additionally, serum biochemical analyses (Fig. S13) revealed no abnormalities in liver function markers (ALT, AST) or kidney function markers (UA, Cr). Overall, these results suggest that PGD/Mg MNs (+) combined with aPD-1/Mg MNs treatment has good biocompatibility and long-term safety.

### 2.6 Systemic immune modulation assessment

**Fig. 6:**
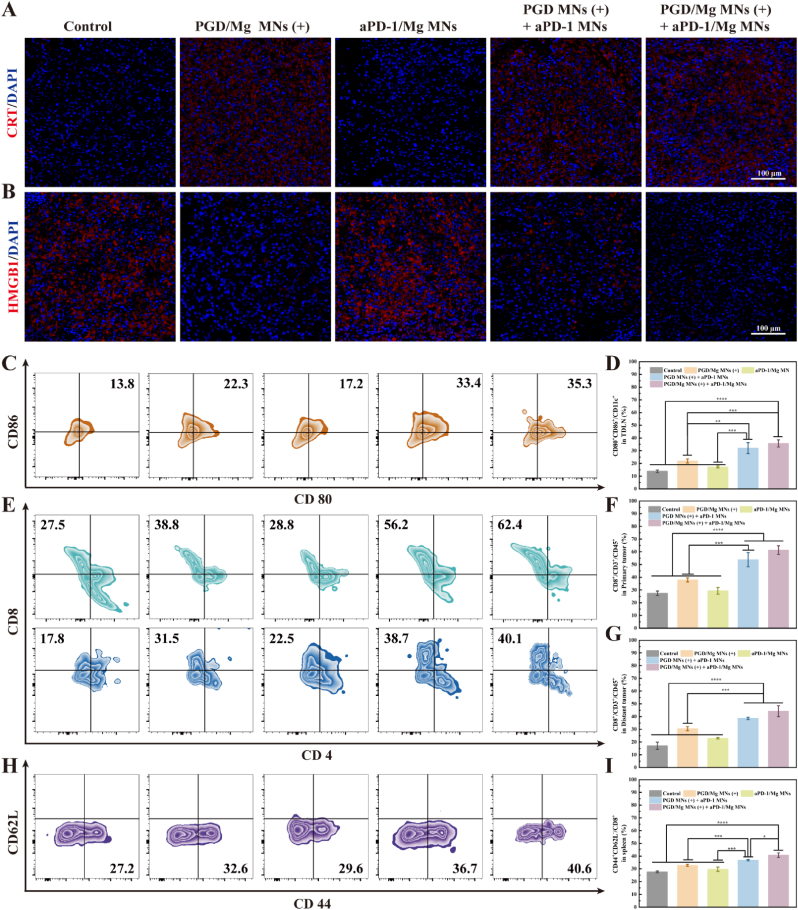
Systemic immune modulation assessment. immunofluorescence staining of (A) CRT (DAPI nuclear stain, blue; CRT, red) and (B) HMGB1 (DAPI nuclear stain, blue; HMGB1, red) in tumor tissues extracted from mice on day 12. (C) Representative flow cytometry images of DC cells (CD80^+^CD86^+^/CD11c^+^) in mouse TDLN and (D) statistical analysis (n = 4). (E) Representative flow cytometry images of CD8^+^ T cells (CD8^+^/CD3^+^/CD45^+^) in primary and distal tumors of mice, and (F–G) statistical analysis. (n = 4) (H) Representative flow cytometry images of memory T cells (CD44^+^CD62L^−^/CD8^+^/CD3^+^) in the spleens of mice, and (I) statistical analysis. (n = 4).

*In vitro* experiments further confirmed that PGD MNs could induce ICD under NIR irradiation. Fluorescence staining results showed that, compared to the untreated group, the expression of CRT in primary and distal tumors was significantly increased in the PGD/Mg MNs (+) treatment group, while the expression of HMGB1 was significantly reduced, indicating that ICD occurred *in vivo*, which may further activate the antitumor immune response (Fig. 6A and B). Mature DCs typically express high levels of CD80^+^/CD86^+^ co-stimulatory molecules on their surface. Flow cytometric analysis demonstrated a marked elevation in dendritic cell maturation (CD86^+^CD80^+^/CD11c^+^) within the TDLN and spleen of mice receiving combined PGD/Mg MNs (+) and aPD-1/Mg MNs therapy (Fig. 6B, C and Fig. S14A and B). In contrast, untreated mice and those treated solely with aPD-1/Mg MNs showed negligible DC maturation. These findings indicate that the photothermal effect mediated by PGD/Mg MNs effectively promotes DC maturation, subsequently enhancing DAMP activation and initiating robust T cell-mediated antitumor immune responses. Moreover, a significant increase in the proportion of CD8^+^ T cells was observed in both primary and distal tumors of mice treated with PGD/Mg MNs (+), indicating the successful induction of a robust immune response. In the PGD/Mg MNs (+) combined with aPD-1/Mg MNs treatment group, the proportion of CD8^+^ T cells further increased. These findings indicate that the combination therapy, by blocking the PD-1/PD-L1 pathway, facilitates effective T cell infiltration while simultaneously mitigating immune evasion within the TME (Fig. 6D–F). Compared to other treatment groups, mice in the combination therapy group exhibited more significant T cell proliferation in the spleen (Fig. S14C and D), further indicating successful activation of antitumor immunity. As illustrated in Fig. 6G and H, the population of effector memory T (T_EM_) cells in the spleens of mice receiving the combined treatment of PGD/Mg MNs (+) and aPD-1/Mg MNs was markedly elevated compared to that in the control group. The higher number of T_EM_ cells indicates long-lasting antitumor efficiency, suggesting that the combined treatment with PGD/Mg MNs (+) and aPD-1/Mg MNs enhances the long-term antitumor capacity and extends survival. Based on our experimental findings, we propose that PGD/Mg MNs (+)-mediated PTT effectively initiates ICD in tumor cells, subsequently activating DAMPs. This cascade significantly enhances dendritic cell maturation and facilitates efficient antigen presentation within tumor-draining lymph nodes. Furthermore, the combination with aPD-1/Mg MNs synergistically increases T cell infiltration, triggering robust T cell-mediated immune responses and markedly amplifying therapeutic antitumor efficacy.

**Fig. 7:**
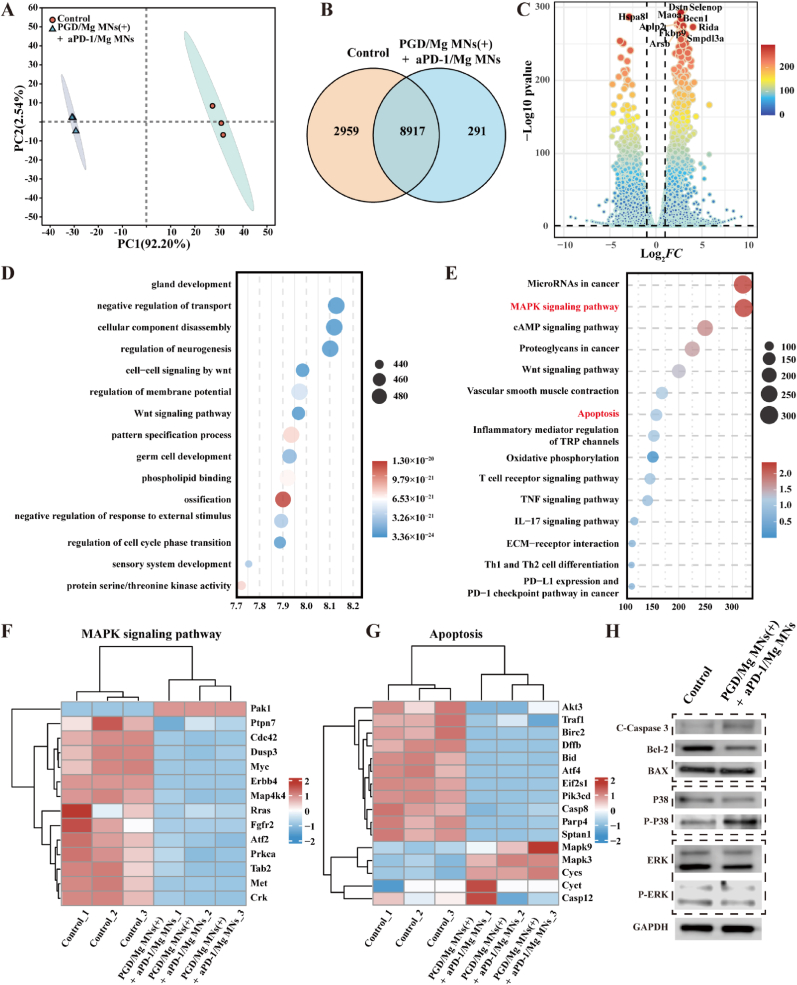
Transcriptomic sequencing of the PGD/Mg MNs (+) and aPD-1/Mg MNs groups after 12 days of treatment compared to the control group. (A) Principal Component Analysis (PCA) scatter plot of the treatment and control groups. (B) Venn diagram of differentially expressed genes between the treatment and control groups. (C) Volcano plot of differentially expressed genes between the treatment and control groups. (D) GO enrichment analysis and (E) KEGG pathway enrichment analysis of differentially expressed genes between the treatment and control groups. Heatmap of differentially expressed genes (DEGs) in the (F) MAPK signaling pathway and (G) Apoptosis pathway. (H) Western blot analysis of proteins in B16 tumor tissues from the treatment and control groups based on the pathways in (F, G). (n = 3).

To further elucidate the potential biological mechanisms of PGD/Mg MNs (+) combined with aPD-1/Mg MNs treatment, RNA sequencing analysis was performed on tumor tissues from the control group and the PGD/Mg MNs (+) + aPD-1/Mg MNs group. Quality control assessments confirmed the consistency of sequencing depth across samples, revealing significant differences between the two groups (Fig. 7A). Moreover, a high degree of overlap in the sequenced genes was observed across different groups (Fig. 7B), indicating robust comparability of the datasets. A total of 12,167 genes were transcribed in the collected tumors, with 2959 genes in the PBS group and 291 genes in the PGD/Mg MNs (+) + aPD-1/Mg MNs group being uniquely transcribed. Analysis of the differentially expressed genes between the groups revealed that in the tumors treated with PGD/Mg MNs (+) + aPD-1/Mg MNs, 4070 genes were upregulated and 4096 genes were downregulated (Fig. 7C and S16).

To assess the antitumor immune response, we further screened immune-related genes, particularly those related to “regulation of immune system processes” and “regulation of apoptotic processes.” GO analysis indicated that the PGD/Mg MNs (+) + aPD-1/Mg MNs group significantly affected the regulation of Negative regulation of transport, Cellular component disassembly, Wnt signaling pathway, and Regulation of membrane potential (Fig. 7D). KEGG pathway analysis demonstrated that the PGD/Mg MNs (+) combined with aPD-1/Mg MNs treatment positively modulated multiple immune response-associated pathways, including the T cell receptor signaling pathway, TNF signaling pathway, and IL-17 signaling pathway. This regulation elicited a robust immune response and effectively promoted CTL activation, aligning well with the findings from *in vivo* experiments. Additionally, pathways related to apoptosis, including the MAPK signaling pathway, cAMP signaling pathway, and apoptosis, were induced (Fig. 7E). In both the MAPK and apoptosis pathways, notable variations were detected in the expression levels of multiple genes associated with cell proliferation, such as downregulation of Akt3, Birc2, and Pik3cd mRNA levels, upregulation of Mapk9, Mapk3, and Cycs mRNA levels related to apoptosis, and downregulation of Cdc42, Myc, Met, and Crk mRNA levels associated with tumor invasion and metastasis. These findings indicate that the combined treatment of PGD/Mg MNs (+) and aPD-1/Mg MNs effectively promotes tumor cell apoptosis while simultaneously suppressing tumor cell invasion and metastasis (Fig. 7F, G and S17A, B).

To further confirm the regulation of apoptosis pathways by PGD/Mg MNs (+) + aPD-1/Mg MNs treatment, Western Blot experiments were performed to assess its impact on the MAPK and apoptosis pathways. The results showed upregulation of Cleaved-Caspase3 and BAX proteins and downregulation of Bcl-2 protein in the apoptosis pathway, indicating activation of the apoptosis pathway, while the upregulation of P-P38 and downregulation of P-ERK in the MAPK pathway indicated activation of the P38 MAPK pathway (Fig. 7H and S18). In conclusion, these results suggest that PGD/Mg MNs (+) combined with aPD-1/Mg MNs treatment can effectively modulate the tumor immune microenvironment, significantly enhancing the efficacy of antitumor immunotherapy.

## 3 Conclusion

In conclusion, we developed a novel gas-propelled microneedle system integrating germanium nanosheets (Ge NSs) and magnesium (Mg) microparticles, which acts as a gas motor. This design enables significantly enhanced drug penetration depth and diffusion range compared to conventional dissolvable microneedles. The Ge NSs provide a strong photothermal effect and high drug-loading capacity, while Mg microparticles generate hydrogen bubbles in situ to promote deeper tissue delivery. By utilising the synergistic effects of photothermal therapy (PTT) and immune checkpoint blockade (ICB), the therapeutic efficacy of tumors was significantly improved. The gas-driven microneedles facilitate deep penetration of PGD NSs and NIR-triggered DOX release, effectively inducing ICD of tumor cells and activating DAMPs. Consequently, this stimulates DCs maturation and initiates a systemic T-cell-mediated immune response. The combination with a PD-1/Mg MNs further inhibits tumor immune escape mechanisms, enhancing CD8^+^ T cell infiltration into the tumor microenvironment and achieving systemic immunity capable of suppressing distant tumor growth solely through localized microneedle administration. Additionally, RNA sequencing and KEGG pathway analyses revealed that this combined therapy positively regulates multiple immune-related and apoptosis-related pathways, providing novel biological insights into immunotherapy mechanisms. Although microneedle technology holds considerable clinical promise for painless melanoma treatment, its limited penetration depth and lack of effectiveness against distant metastases significantly compromise its clinical value. Therefore, this study proposes a novel approach to painlessly treat deep-seated and distant melanoma lesions, greatly enhancing the clinical applicability and value of microneedle technology.

## 4 Materials and methods

### 4.1 Synthesis of Ge NSs

Germanium powder (Naiounano, China) was dispersed in an isopropanol solution and subjected to ultrasonic exfoliation using a 5 mm probe Ultrasonic Cell Crusher (SCIENTZ, SCIENTZ-ⅡD, China) at 600 W for 24 h while maintaining an ice bath to prevent overheating. The resulting suspension was sequentially centrifuged (Beckman Coulter, Allegra64R Centrifuge, USA) at 3000 rpm for 20 min to collect the supernatant, followed by additional centrifugation steps at 4000 rpm and 5000 rpm for 10 min each to isolate the precipitate. The collected precipitate was subsequently freeze-dried using a lyophilizer (Christ, Alpha 2–4 LDplus, Germany), and the obtained Ge NSs were stored at 4 °C for further use.

### 4.2 Synthesis of PG NSs

DSPE-PEG (5k, China) and Ge NSs were individually dispersed in equal volumes of chloroform (CHCl_3_) (Guangzhou Chemical Reagent Factory, China). The two solutions were then combined and subjected to sonication for 20 min in an ice bath to facilitate uniform dispersion. Following sonication, chloroform was completely removed via vacuum rotary evaporation (IKA, RV10, Germany). The resulting solid residue was resuspended in deionized water and subsequently centrifuged at 12,000 rpm for 10 min to separate the supernatant. The collected precipitate was thoroughly washed three times with deionized water, freeze-dried, and the obtained PG NSs were stored at 4 °C for further use.

### 4.3 Synthesis of PGD NSs

DOX (Aladdin, China) was completely dissolved in PBS (pH 7.4, Aladdin, China) at a mass ratio of 10:1, followed by the addition of PG NSs. The resulting mixture was continuously stirred under dark conditions at 4 °C for 24 h to facilitate drug loading. Subsequently, the suspension was centrifuged at 10,000 rpm for 30 min, and the supernatant was carefully collected for further analysis. The obtained precipitate was thoroughly washed three times with deionized water, freeze-dried, and the final PGD NSs were obtained for subsequent experiments.

### 4.4 Synthesis of PGD/mg MNs and a PD-1/mg MNs

Magnesium metal particles (Naiounano, China) were dispersed in isopropanol (Macklin, China) and the solution was pipetted into a microneedle mold (Micropoint Technologies Pte Ltd, Singapore). Vacuum was applied to ensure the magnesium particles filled the very tips of the needles. PGD NSs and anti PD-1 peptide powder (MCE, USA) were each dispersed in deionized water and added dropwise into a microneedle mold under vacuum, allowing the PGD NSs or PD-1 peptide to fill the tips of the needles. Finally, a 10 % dextran solution (kDa: 70000, J&K Scientific, China) was added to the mold, vacuum applied, and dried to obtain PGD/Mg MNs and aPD-1/Mg MNs.

### 4.5 Drug loading and *in vitro* drug release characterization

Following the synthesis of PGD NSs, the supernatant obtained from centrifugation was analyzed to determine the DOX content using a UV–vis–NIR spectrophotometer (Shimadzu Corporation, UV-2550, Japan) by measuring absorbance at 490 nm. The drug loading efficiency was then quantified based on the following formula:

$$Drug\;loading\;\left(\%w/w\right)=\frac{\left(Amount\;of\;DOX\;fed-DOX\;content\;in\;supernatant\right)}{Total\;mass}\times 100\%$$

Equal amounts of PGD NSs were placed into two dialysis bags (14 kDa) and immersed in PBS solutions at different pH levels (7.4 and 5.0). At predetermined time intervals (2, 4, 6, 12, 24, 48, 72, and 96 h), the DOX concentration was quantified by measuring absorbance at 490 nm using a UV–vis–NIR spectrophotometer. Following each measurement, 100 mL of fresh PBS (pH 5.0 or 7.4) was added to maintain a constant solution volume. To assess photothermally-triggered drug release, 2 mL of PGD NSs dispersed in PBS (pH = 5.0) was subjected to identical conditions and irradiated with an 808 nm NIR laser (Changchun Laser Technology Co. LTD, ADR-1805, China) at designated time points (0, 1, 4, and 6 h). Before and after each irradiation, 100 mL of the solution was withdrawn for DOX quantification, and an equivalent volume of PBS was replenished to maintain the total solution volume.

### 4.6 Characterization of photothermal properties of the nanomaterials

PG NSs were prepared at varying concentrations (25, 50, 100, and 200 μg/mL) and exposed to 808 nm NIR laser irradiation at different power densities (0.5, 1, 1.5, and 2 W/cm^2^). The resulting temperature variations were monitored and recorded using an infrared thermal imager (FOTRIC, 225s, China).

Ge NSs and PG NSs dispersions were placed in a 48-well plate with pure water serving as the control. They were irradiated with an 808 nm NIR laser for 5 min, followed by cessation of irradiation to allow the dispersion temperatures to return to baseline. This procedure was repeated five times, with a thermocouple thermometer (TES, TES1310, Taiwan China) recording the real-time temperatures of the materials every 10 s to plot the heating-cooling cycle curves.

### 4.7 Cell cytotoxicity assay

The cytotoxic effects of hydrogel microneedles on mouse melanoma B16 cells were evaluated using a CCK-8 assay kit. B16 cells were seeded in 96-well plates at a density of 5000 cells per well and incubated in a humidified CO_2_ incubator for 24 h to allow cell adhesion. Different nanomaterial dispersions were sterilized by exposure to UV light for 0.5 h inside a biosafety cabinet. After removing the old medium from the 96-well plates, 100 μL of PG MNs or PGD MNs was added to each well. One group was irradiated with an 808 nm near-infrared (NIR) laser for 5 min, while the other group received no additional treatment. The plates were then further incubated for 24 h. After incubation, the medium was removed, and each well was washed three times with sterile PBS. Next, 100 μL of culture medium containing CCK-8 reagent was added to each well and incubated for an additional 1 h. Finally, the optical density (OD) was measured at 450 nm using a microplate reader.

### 4.8 Live/dead cell staining

The cytotoxic effects of hydrogel microneedles on B16 cells were further visualized using an inverted fluorescence microscope and a live/dead cell staining kit to intuitively assess their antitumor performance *in vitro*. B16 cells were first seeded into 96-well plates, followed by the addition of PG NSs or PGD NSs. One group was subjected to 808 nm NIR irradiation for 5 min, while the other was left untreated. After a further 24-h incubation, cells were stained using a live/dead cell staining kit, and fluorescence imaging was performed with an inverted fluorescence microscope.

### 4.9 Mitochondrial membrane potential assay

B16 cells were cultured in confocal dishes until reaching approximately 70 % confluence. Cells were then treated with PG MNs or PGD MNs and incubated for 4 h. One group received 808 nm NIR laser irradiation, while the other did not. Additionally, a control group treated with fresh medium alone was also subjected to 808 nm NIR irradiation. After a further 1-h incubation, all groups were stained with JC-1 dye for 30 min. Cells were washed twice with PBS before fluorescence imaging was conducted using a confocal laser scanning microscope.

### 4.10 Cellular uptake assay

B16 cells were seeded into confocal dishes and allowed to reach approximately 70 % confluence. Half of the cells were incubated with PGD MNs. The other half underwent the same treatment, with the addition of 808 nm NIR irradiation for 5 min. After 6 h of incubation, cells were washed twice with fresh PBS and stained with DAPI for 10 min. Samples were then observed using a confocal laser scanning microscope.

### 4.11 Animal experimental model

The *in vivo* tumor model was established in strict compliance with the guidelines set forth by the Institutional Animal Care and Use Committee (IACUC, 20240802-001), which approved all animal experimental protocols described in this study. To generate an orthotopic tumor model, female C57BL/6 mice (6–8 weeks old) were anesthetized by intraperitoneal injection of pentobarbital sodium at a dose of 50 mg/kg. Subsequently, a subcutaneous orthotopic tumor model was established by injecting 3 × 10^5^ B16 cells into the right dorsal region. Three days post-inoculation, a secondary distal tumor model was similarly established on the left dorsal flank. Treatment was initiated once the volume of the orthotopic tumor reached approximately 100 mm^3^. Tumor volume was determined using the formula:

$$Tumor\;Volume=\frac{Length\times {Width}^{2}}{2}$$

Throughout the treatment period, tumor dimensions, including length and width, were measured daily using vernier caliper (Deli, China), while the real-time body weight of the nude mice was recorded with an electronic scale. After 12 days of treatment, the mice were euthanized. The excised tumors were meticulously dissected, photographed, and weighed before being either fixed in 4 % paraformaldehyde (Servicebio, China) for histological analysis or stored at −80 °C in an ultra-low temperature freezer (Yaxubio, Merit, China) for subsequent investigations.

### 4.12 Flow cytometry protocol

Lymph nodes, spleens, and tumor tissues were carefully dissected from the mice and transferred to a petri dish, where they were mechanically dissociated in RPMI 1640 medium. For spleen samples, red blood cells were lysed by adding red blood cell lysis buffer, incubating at room temperature for 1–2 min, followed by centrifugation to remove the red-colored supernatant. Tumor tissues were enzymatically digested in RPMI 1640 medium supplemented with 0.01 % DNase I (Roche, Switzerland) and 0.1 % collagenase IV (Sigma, USA), then incubated in a 37 °C shaker for 30–60 min. Following digestion, the tissues were washed with PBS, and the resulting cell suspension was collected. The cells were subsequently centrifuged, resuspended in PBS containing 1 % BSA, and incubated with the appropriate flow cytometry antibodies (BioLegend, USA) on ice for 30 min. After a final centrifugation, the cells were resuspended in 300 μL of PBS and analyzed using a flow cytometer (URIT, BF-710, China).

### 4.13 Western Blot

Tumor tissues were cut into small pieces (3–5 mm^3^) and ground using a grinder. Lysis buffer was added to lyse the cells for 2 h. The tissue homogenate was then sonicated for 60 s in an ice bath using an ultrasonic cell disruptor, followed by centrifugation at 12,000 g and 4 °C for 30 min to collect the supernatant.

Proteins were denatured by boiling at 100 °C. The protein concentration of the samples was determined using a BCA Protein Assay Kit (Beyotime, China), and the volume for each sample was calculated accordingly. SDS-PAGE gels (Solarbio, China) were prepared as per the instructions, and the electrophoresis tank was filled with running buffer (Solarbio, China). Samples and marker (Solarbio, China) were loaded, and electrophoresis was conducted at 200V for 30 min.

The gel was carefully transferred onto filter paper within a transfer cassette, and a methanol-activated PVDF membrane was positioned over the gel before placing the cassette into the transfer tank. Protein transfer was conducted at 250 mA for 30 min. Following transfer, the PVDF membrane was blocked with 5 % skim milk prepared in TBST (Solarbio, China) and incubated on a shaker for 1 h. Subsequently, the membrane was transferred to a primary antibody incubation box and incubated overnight at 4 °C with the designated primary antibodies, including C-Caspase3, Bcl2, BAX, ERK, P38, Goat anti-Mouse IgG, and Goat anti-Rabbit IgG (Proteintech, China), p-ERK (Santa, USA), p38 (Bioss, China), and GAPDH (CST, USA). After primary antibody incubation, the membrane was treated with the corresponding secondary antibody and incubated at room temperature for 1 h. To visualize the protein bands, the PVDF membrane was exposed to ECL substrate (Solarbio, China) and subsequently imaged using a gel imaging system (LICORbio, C-DiGit Blot Scanner, USA).

### 4.14 Statistical analysis

Each experiment was conducted independently at least three times (n ≥ 3), ensuring reproducibility across multiple synthesis batches. Data were expressed as the mean ± standard deviation (SD). Statistical analyses were performed using Origin 2024 software. Group comparisons were evaluated using Student's t-test and one-way ANOVA. Statistical significance was defined as p < 0.05, with significance levels denoted as follows: (∗) for p < 0.05, (∗∗) for p < 0.01, (∗∗∗) for p < 0.001, and (∗∗∗∗) for p < 0.0001.

## CRediT authorship contribution statement

**Chungchi Lee:** Writing – review & editing, Writing – original draft, Visualization, Validation, Methodology, Investigation, Formal analysis, Data curation, Conceptualization. **Shanghui Huang:** Writing – review & editing, Writing – original draft, Visualization, Validation, Methodology, Investigation, Formal analysis, Data curation, Conceptualization. **Huiling Liu:** Writing – review & editing, Visualization, Supervision, Methodology, Investigation, Data curation. **Xinyue He:** Writing – review & editing, Validation, Methodology, Investigation, Data curation. **Yifan Hao:** Writing – review & editing, Validation, Methodology, Investigation, Data curation. **Lizhi Zeng:** Writing – review & editing, Validation. **Yuhan Li:** Writing – review & editing, Validation. **Zijin Lv:** Writing – review & editing, Validation. **Yiyang Xu:** Writing – review & editing, Validation. **Rui Guo:** Writing – review & editing, Writing – original draft, Validation, Supervision, Resources, Funding acquisition, Conceptualization.

## Declaration of competing interest

The authors declare that they have no known competing financial interests or personal relationships that could have appeared to influence the work reported in this paper.

## Data Availability

Data will be made available on request.
