# Supplementary material for: Self-propelled gas nanomotor-integrated microneedles for melanoma therapy: Dual-action in situ eradication and metastatic suppression
Source: Mater Today Bio. 2025 Jul 21;34:102122. doi: 10.1016/j.mtbio.2025.102122 (PMC12311587; doi:10.1016/j.mtbio.2025.102122)
Supplement: Multimedia component 1 [file mmc1.docx]

**Supporting**

**S1.** The hydrodynamic diameter of (A) Ge NSs, (B) PG NSs and (C) PGD NSs. (D) Changes in the hydrodynamic diameter and dispersity of PGD NSs after 24 h of incubation in 10% FBS.

**S2.** XPS spectra of (A) Ge NSs, (B) PG NSs in different binding energy ranges.

**S3.** FT-IR spectra of DOX, and DSPE-PEG.

**S4.** XRD spectrum of Ge NSs, PG NSs, PGD NSs and standard Ge card.

**S5.** Photographic comparison of PG NSs (left) and PGD NSs (right) solutions before and after centrifugation.

**S6.** The temperature variation (ΔT) of PG NSs solutions at different concentrations.

**S7.** Images of MNs, Mg MNs and PGD/Mg MNs.

**S8.** Photothermal images of MNs, Mg MNs, PGD MNs and PGD/Mg MNs under NIR irradiation (808 nm, 1.5 W/cm^2^, 5 min).

**S9.** TEM images of B16 cells after treatment with different materials.

**S10.** *In vivo* photothermal images of Control and PGD/Mg MNs with and without NIR irradiation (808 nm, 1.5 W/cm^2^, 5 min).

**S11.** H&E staining of heart, liver, spleen, lung and kidney tissues extracted from mice on day 12.

**S12.** Routine blood analyses of mice on day 12.

**S13.** Serum biochemical analysis of mice on day 12.

**S14.** (A) Representative flow cytometry images of DC cells (CD80^+^CD86^+^/CD11c^+^) in mouse spleen and (B) statistical analysis. (C) Representative flow cytometry images of CD8^+^ T cells (CD8^+^/CD3^+^) in mouse spleen and (D) statistical analysis.

**S15.** Schematic diagram of the gating strategy used for flow cytometry analysis.

**S16.** The differential gene heatmap of Control and PGD/Mg MNs (+) + aPD-1/Mg MNs.

**S17.** Protein interaction network diagram of differentially expressed genes (DEGs) in MAPK signaling pathway and Apoptosis.

**S18.** Semi-quantitative analysis of Western blot results shown in Fig. 7H.


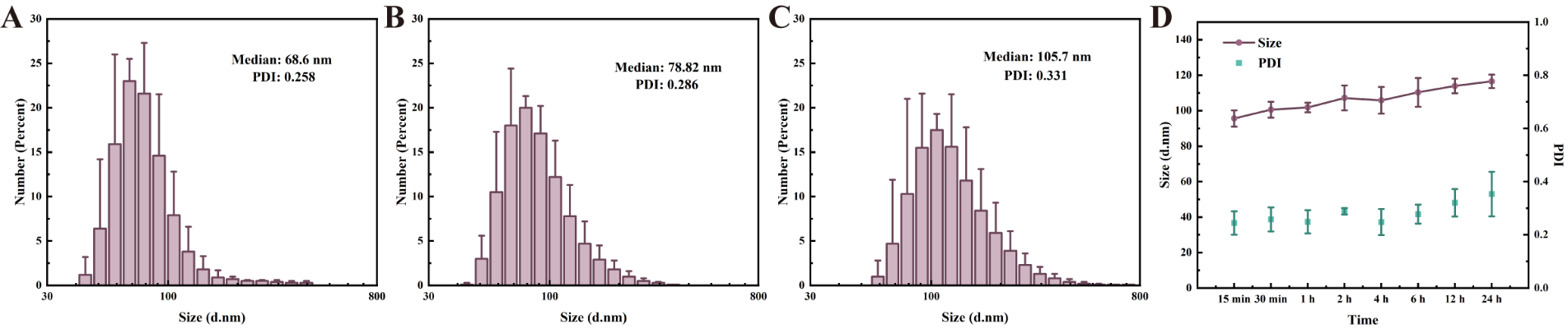


**S1**


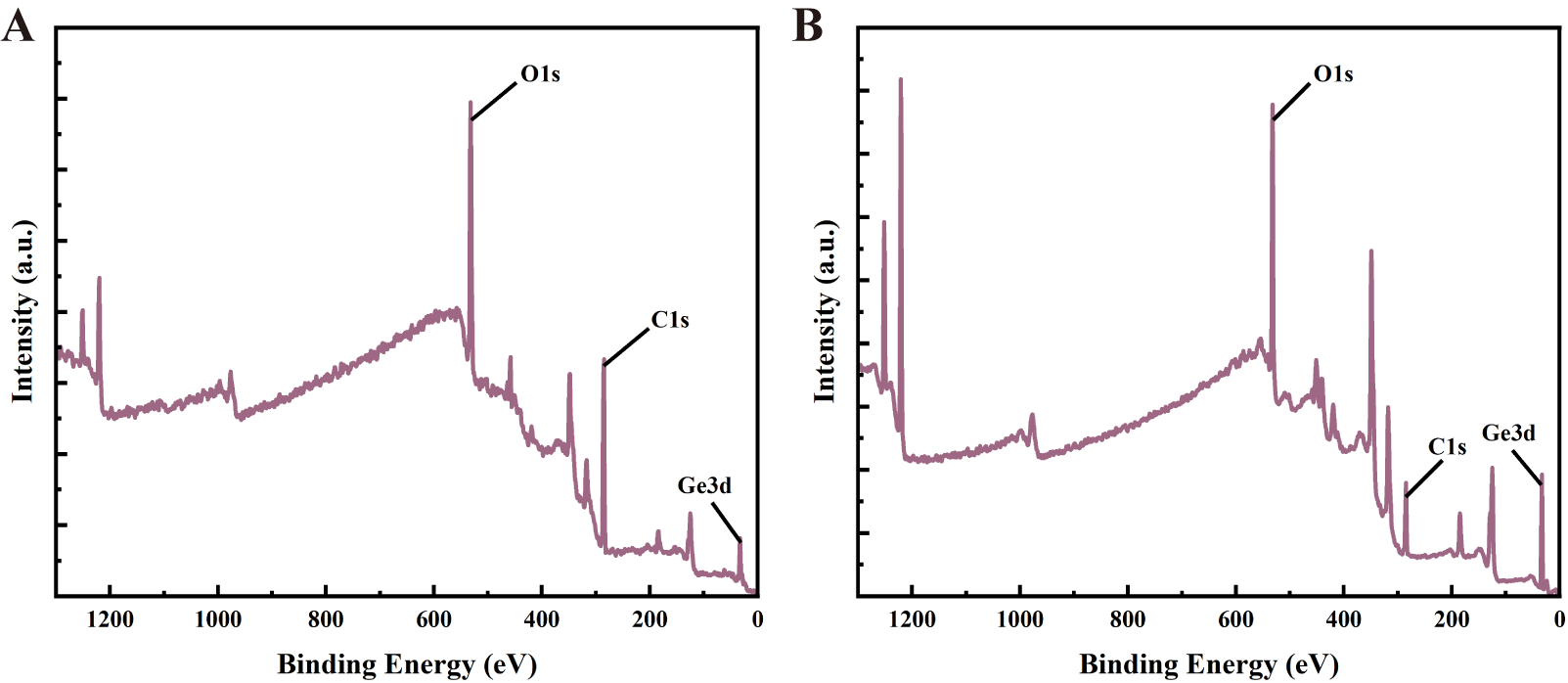


**S2**

**
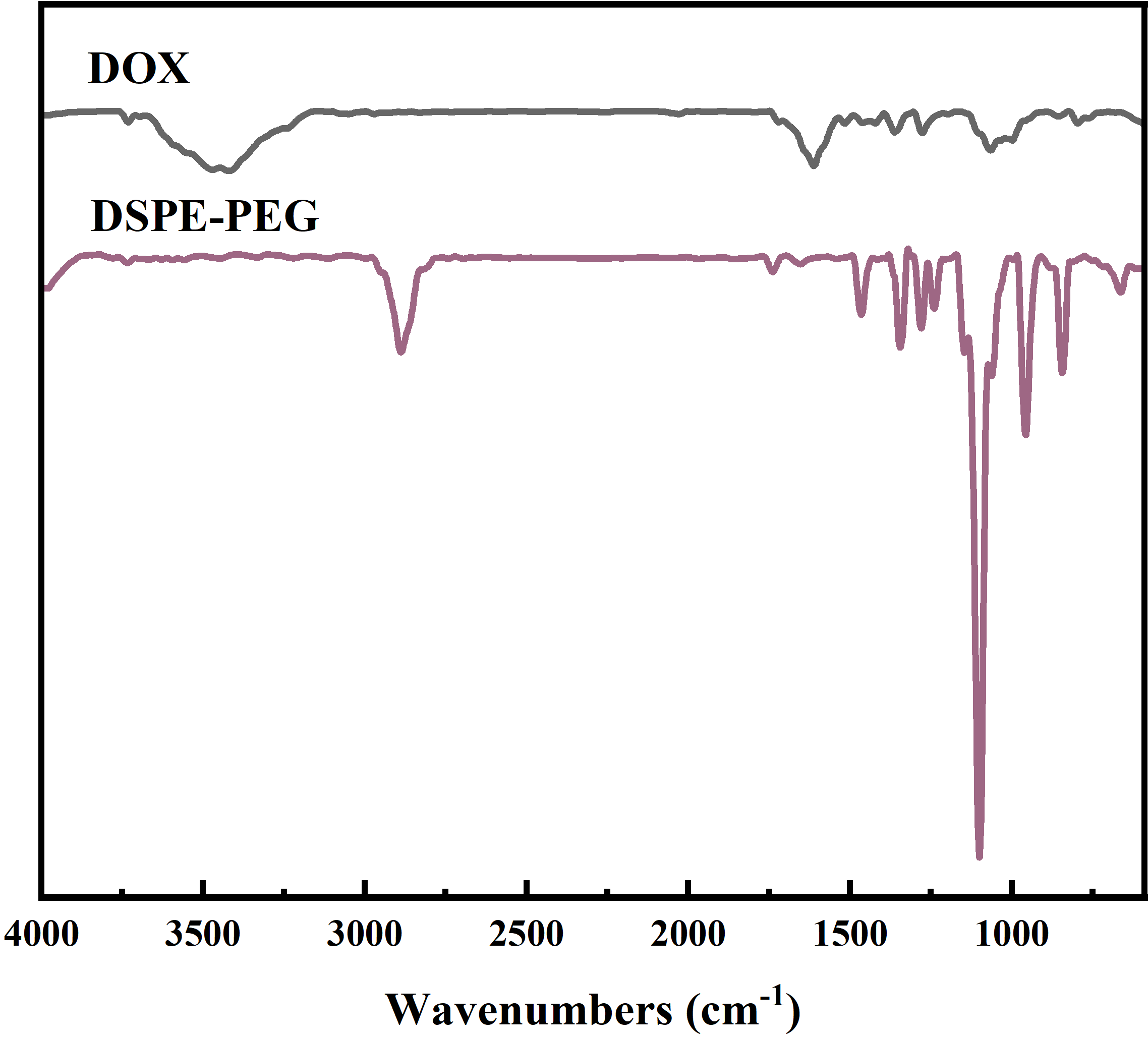
**

**S3**

**
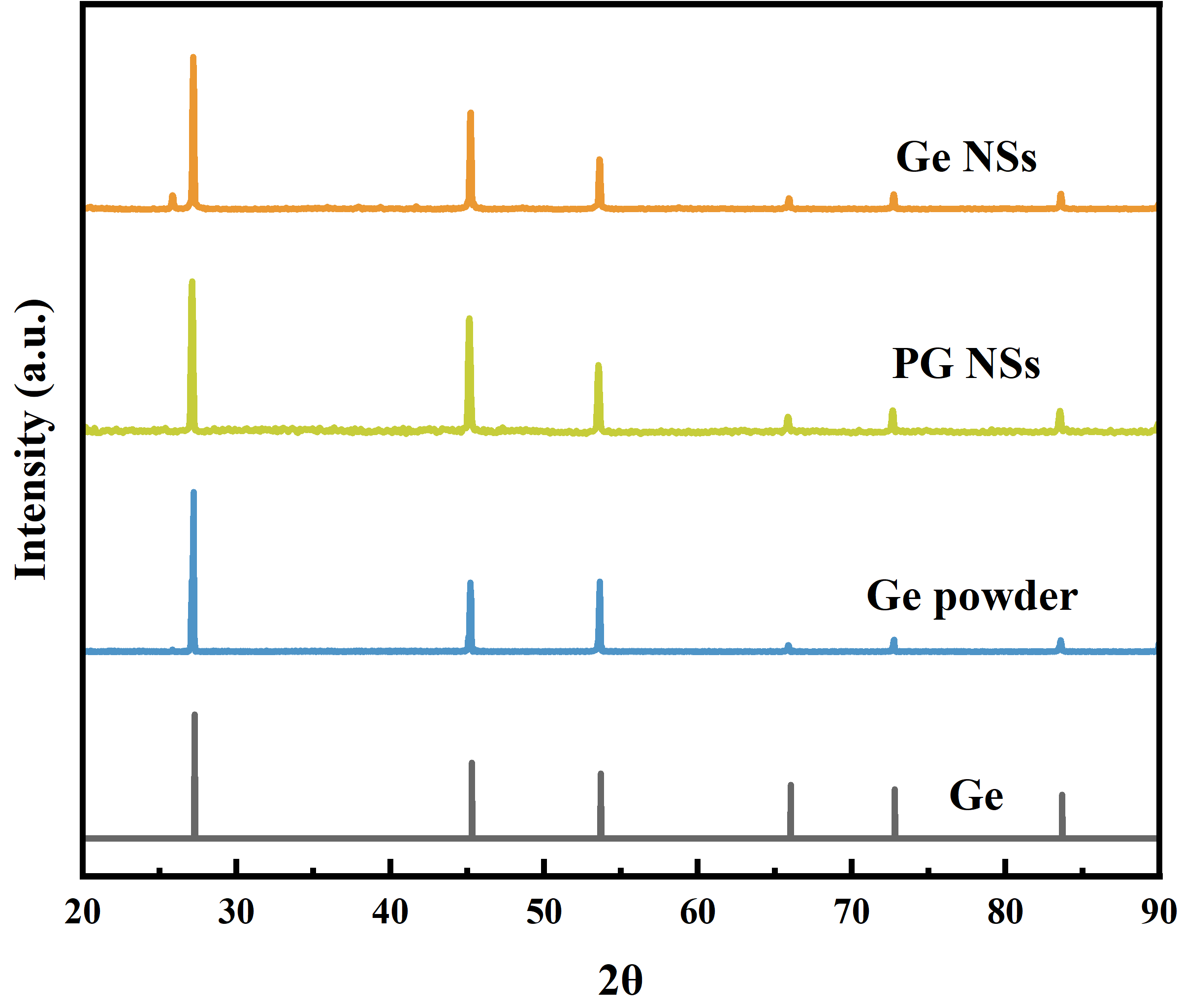
**

**S4**

**
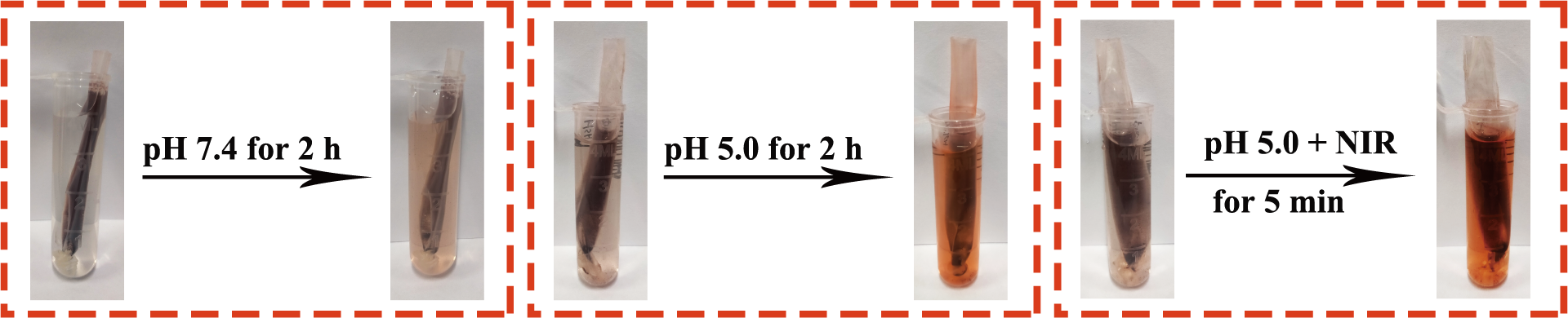
**

**S5**

**
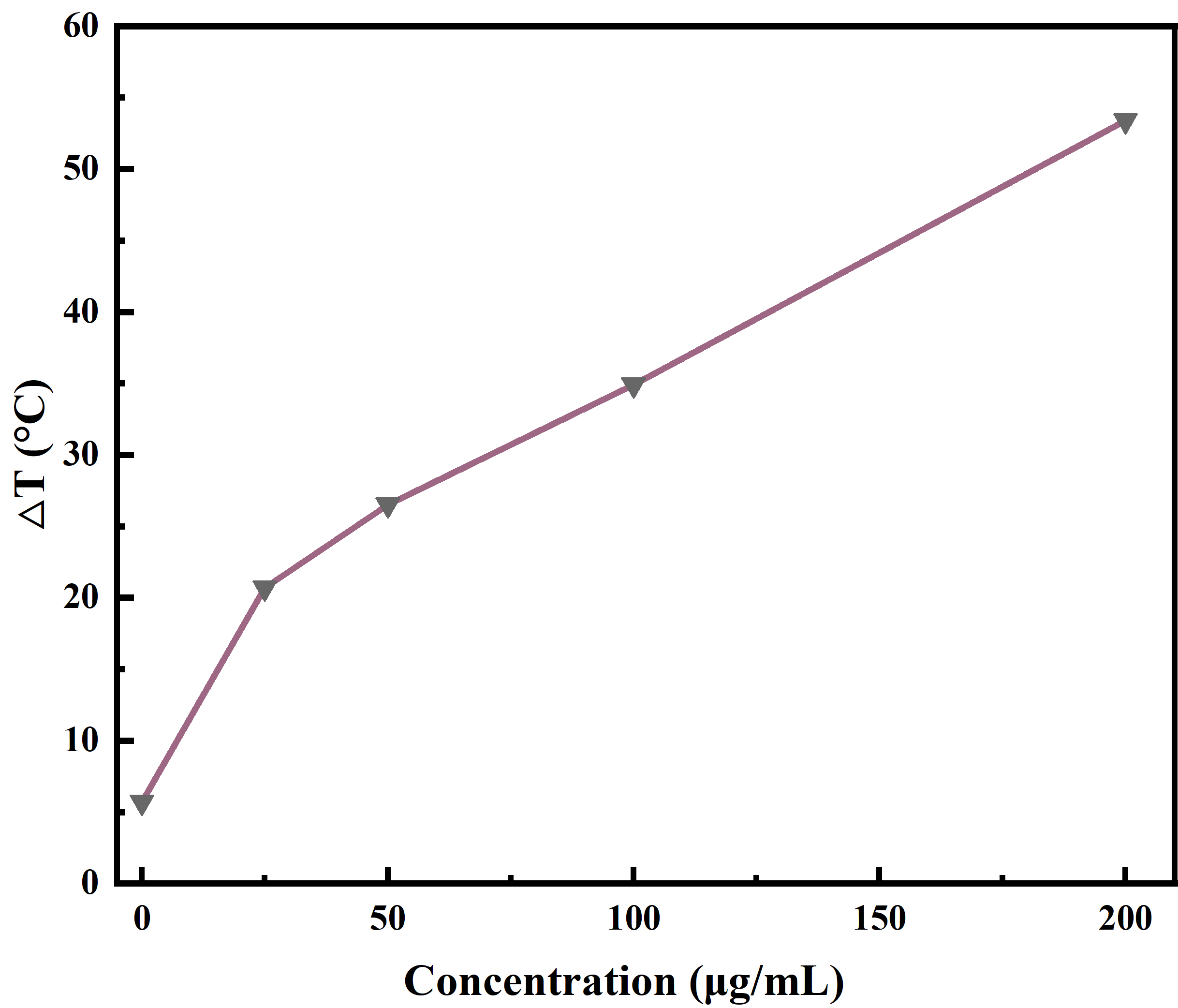
**

**S6**

**
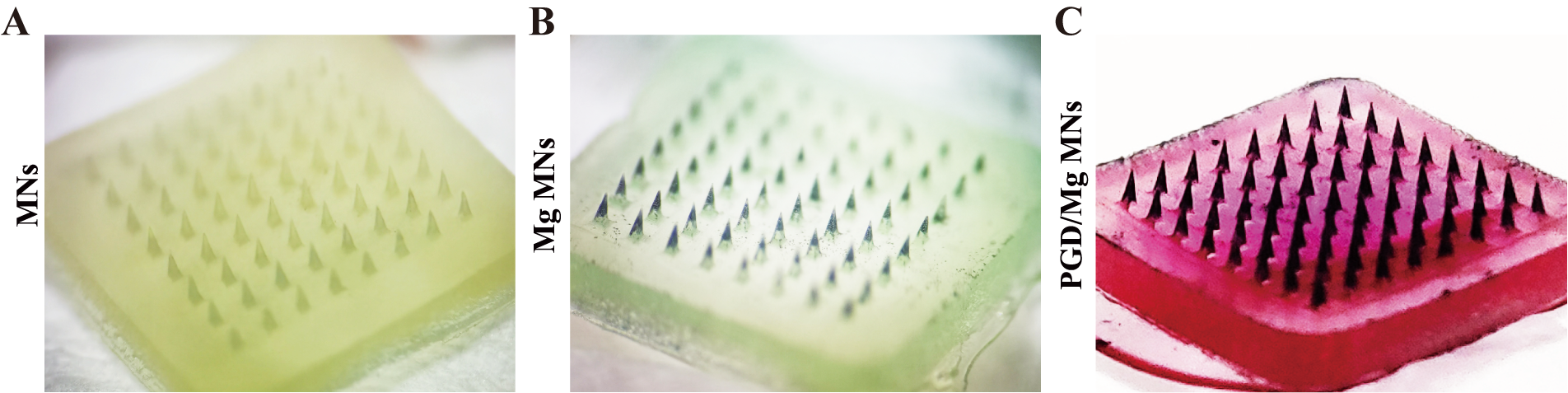
**

**S7**


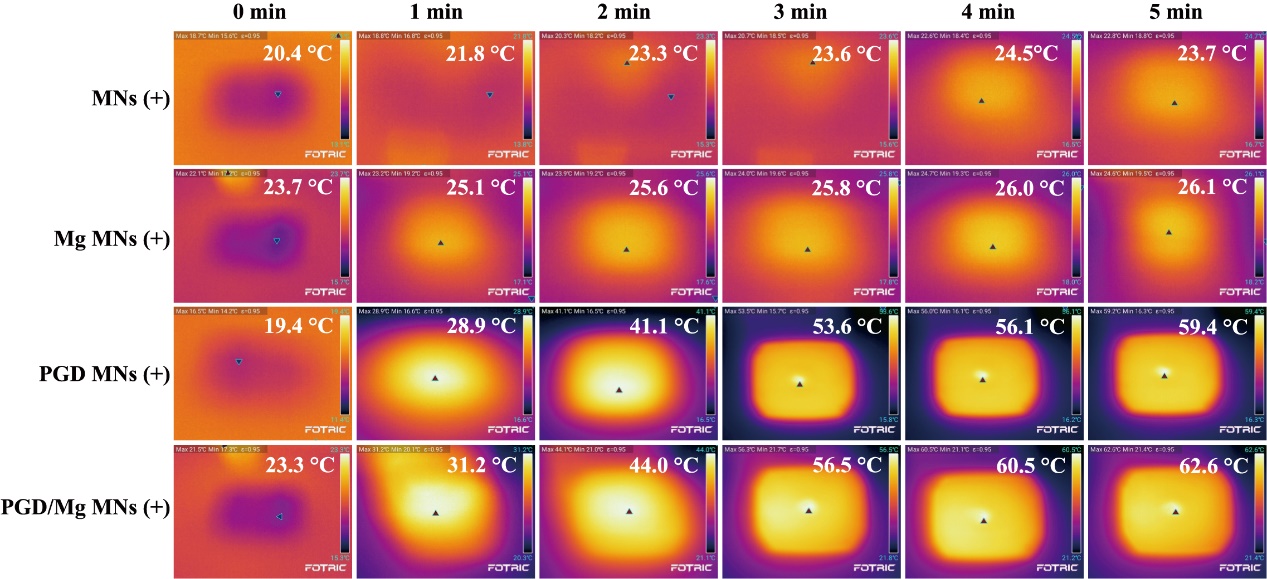


**S8**


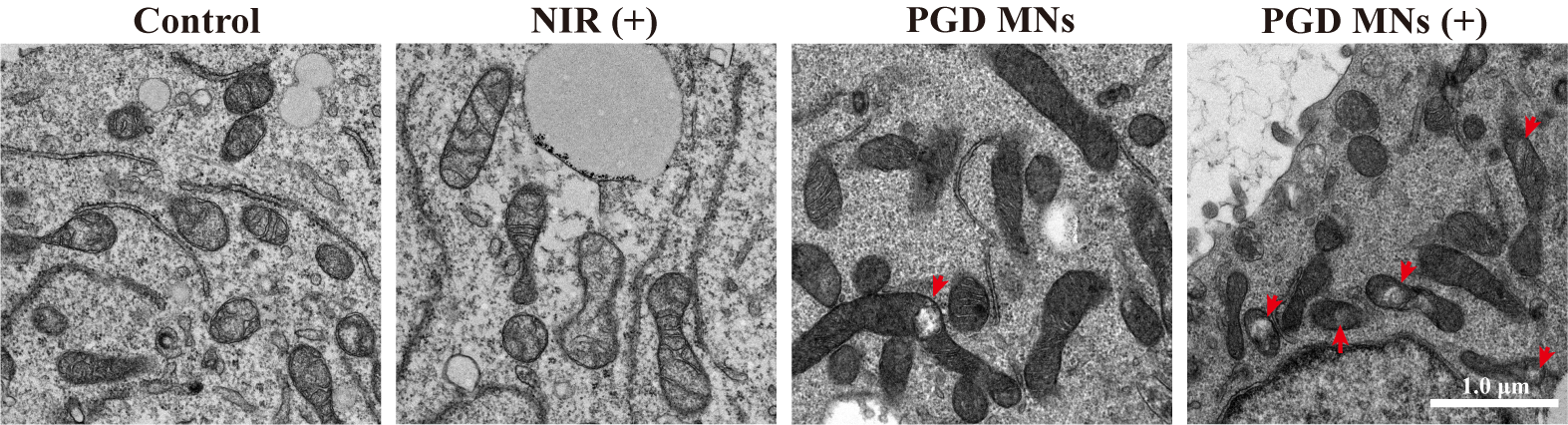


**S9**


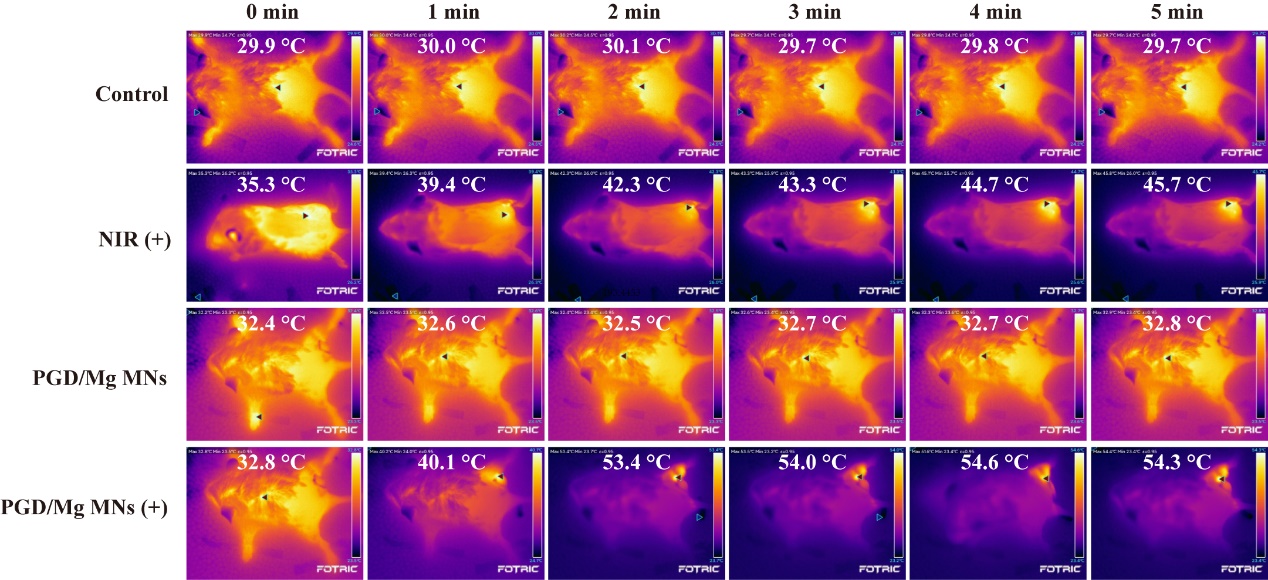


**S10**

**
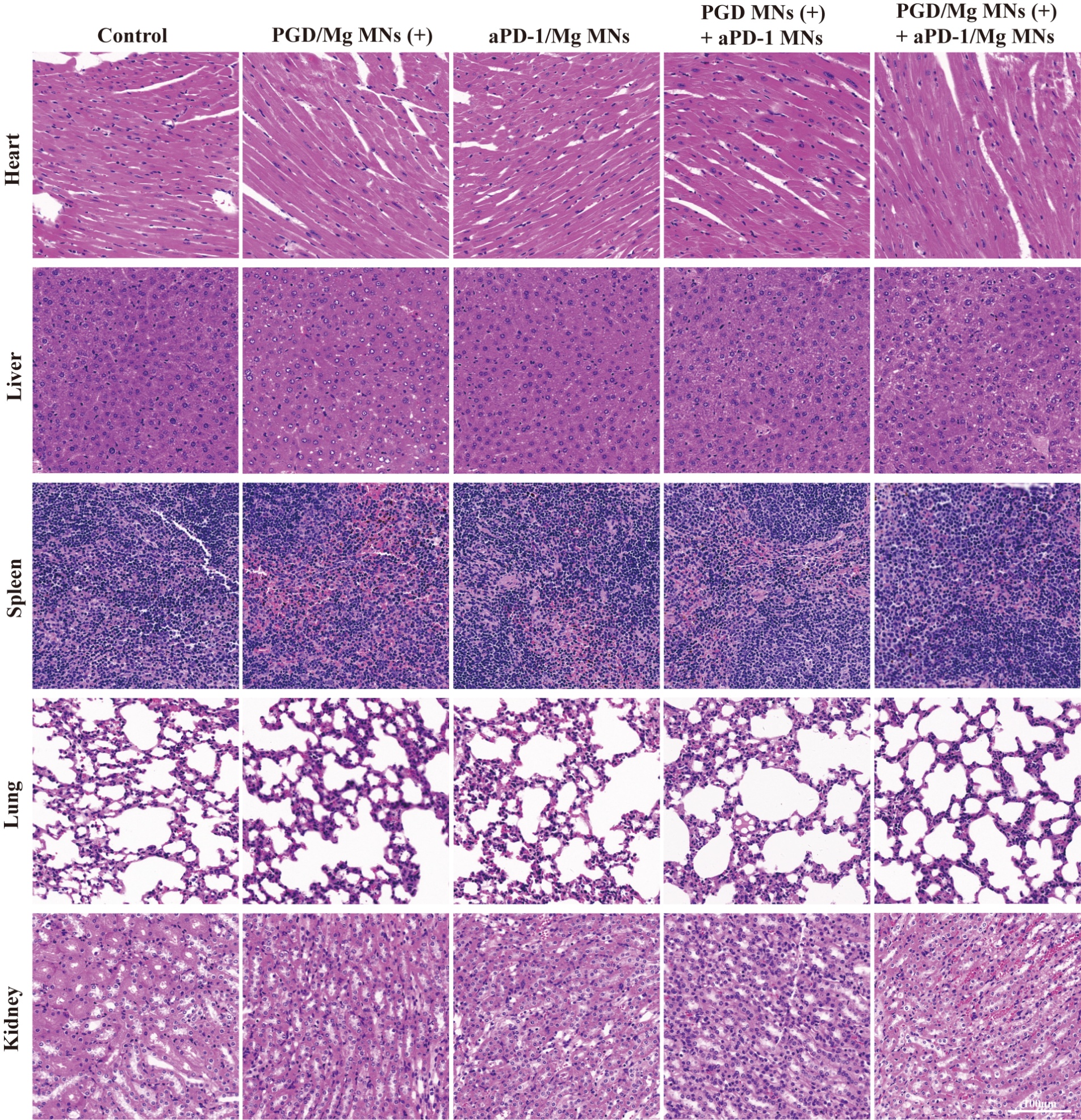
**

**S11**

**
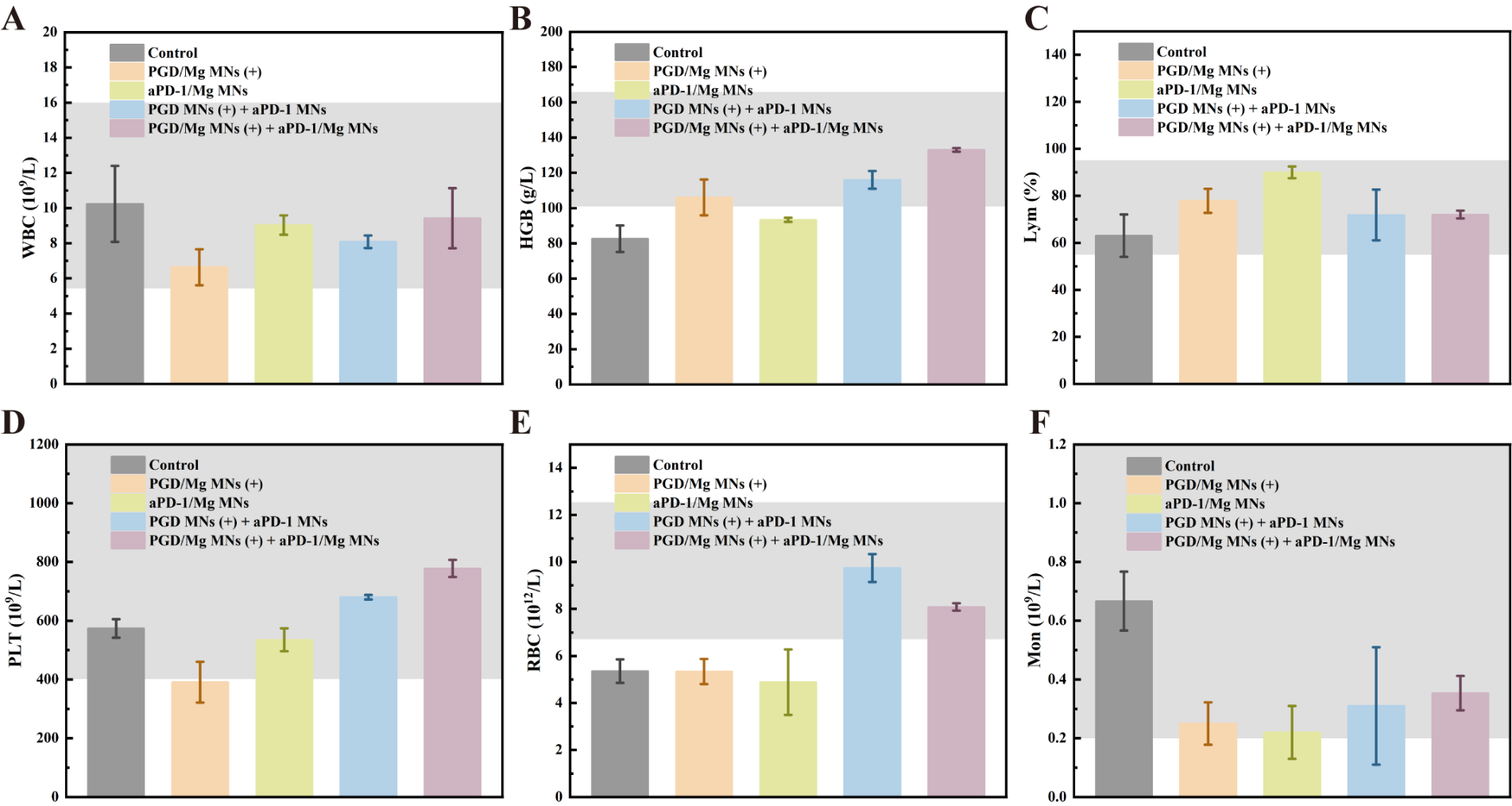
**

**S12**


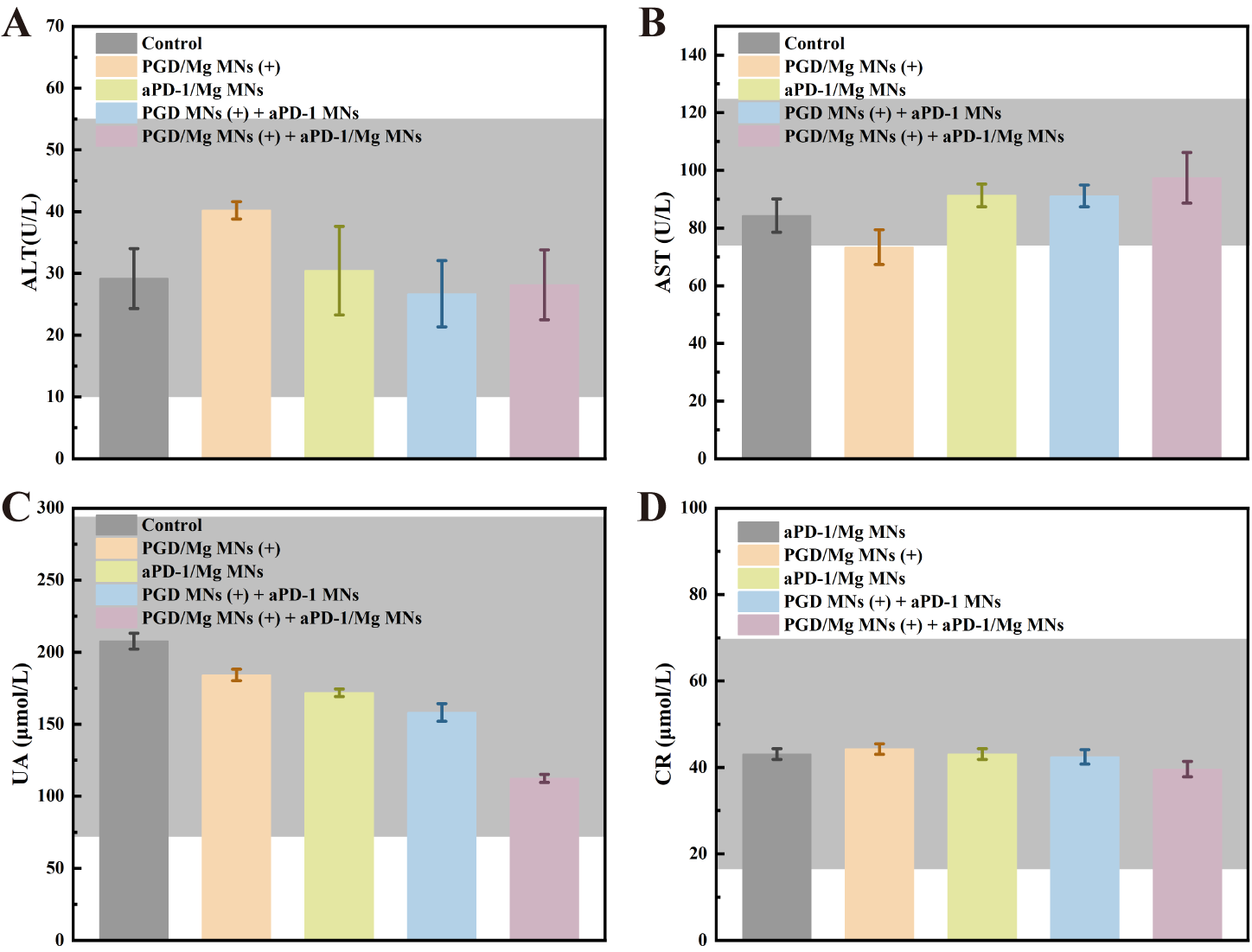


**S13**

**
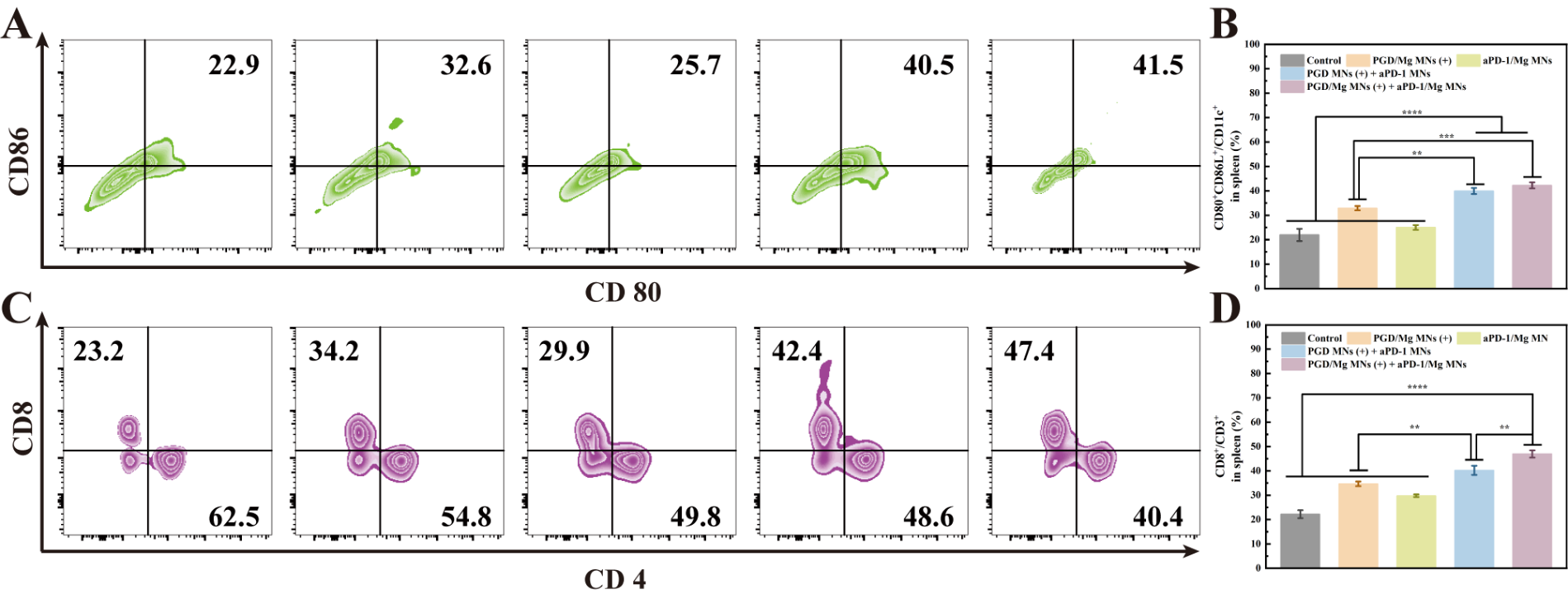
**

**S14**


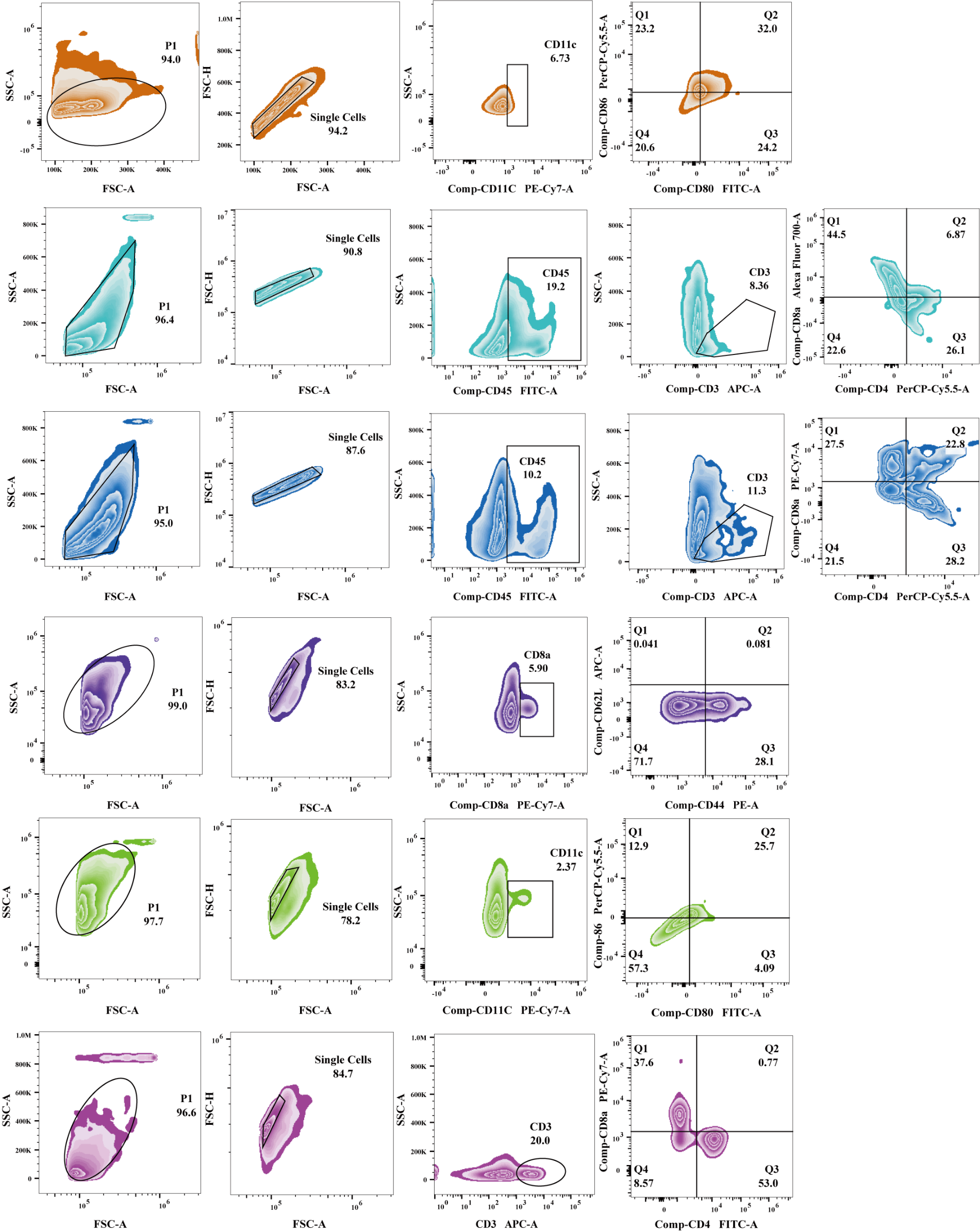


**S15**

**
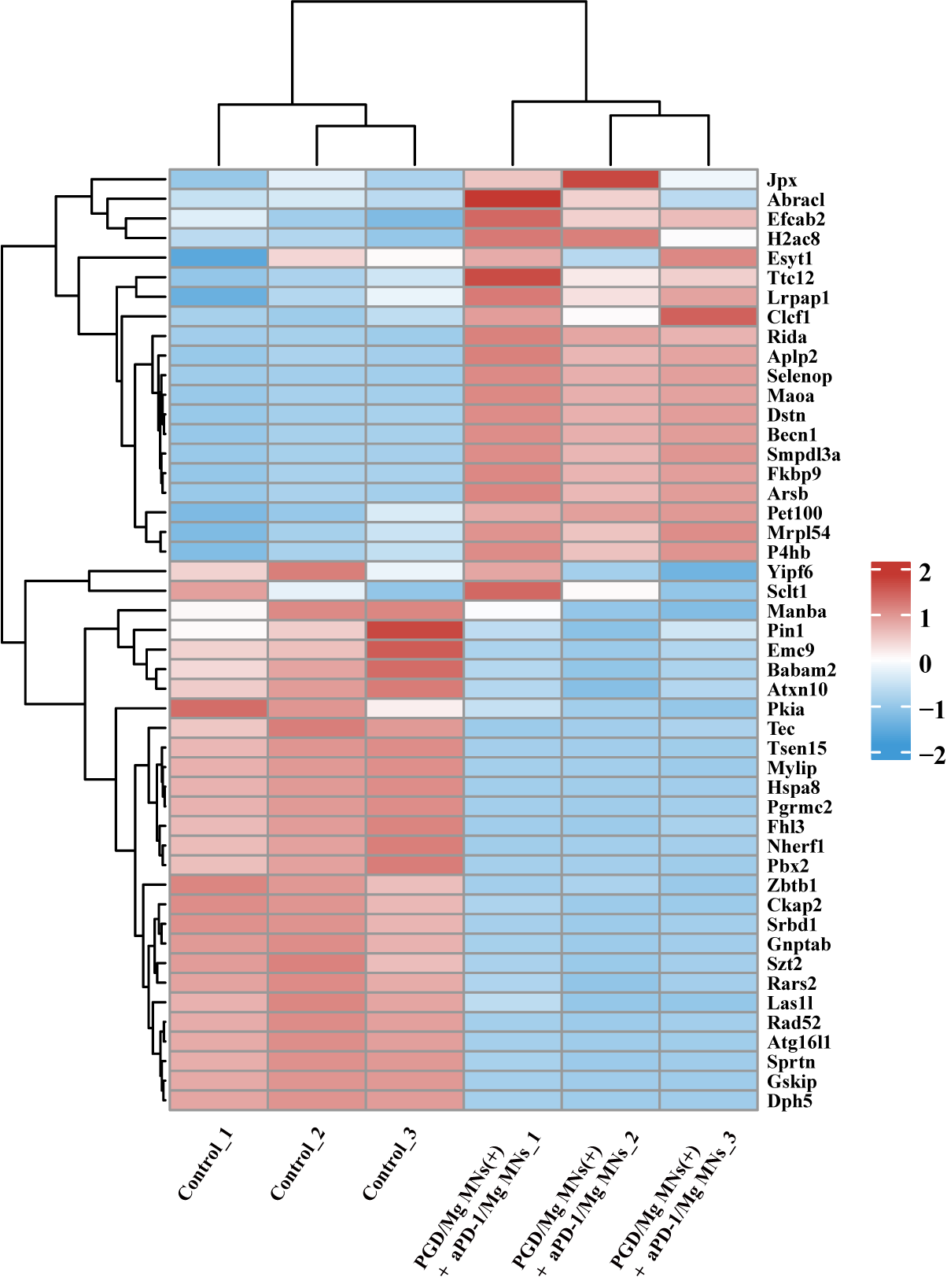
**

**S16**

**
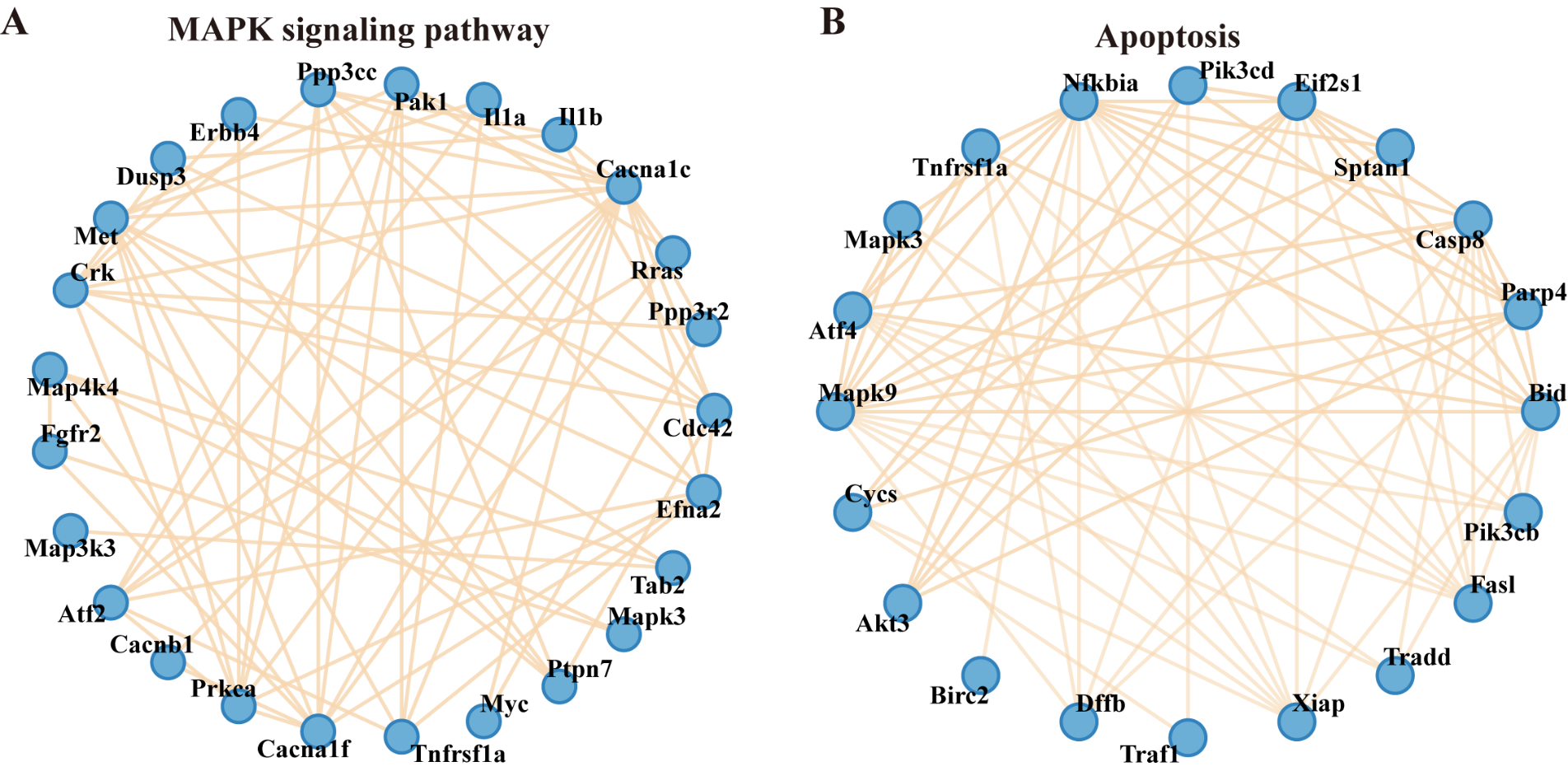
**

**S17**

**
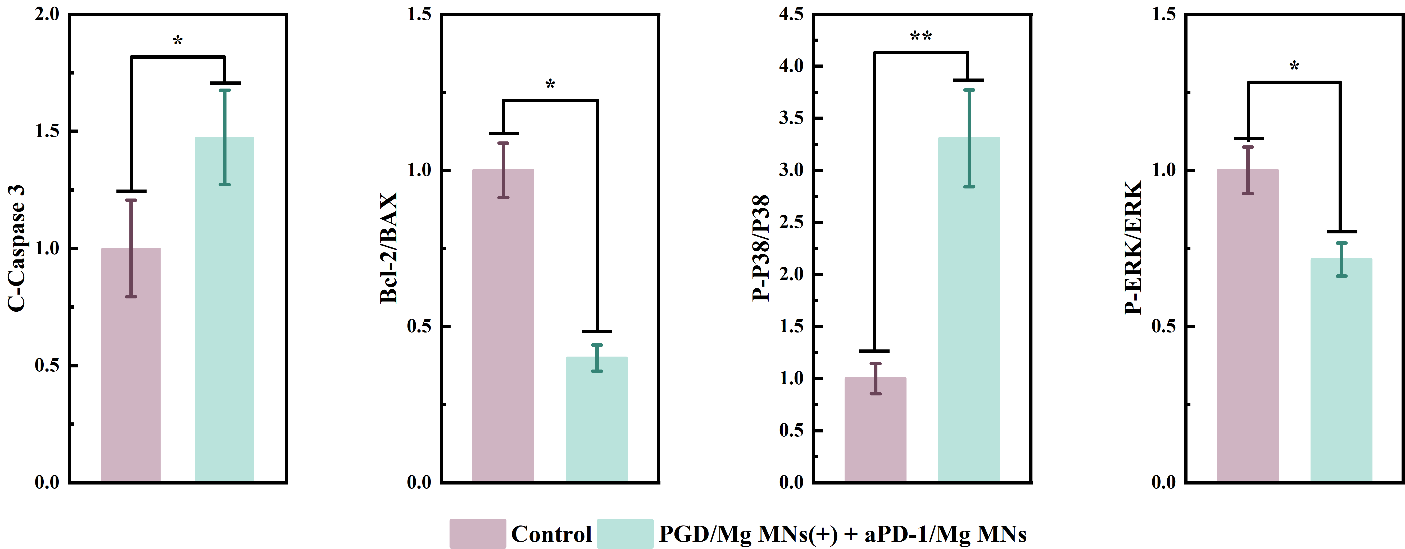
**

**S18**
